# Improved Constraints on Global Methane Emissions and Sinks Using *δ* ^13^C‐CH_4_

**DOI:** 10.1029/2021GB007000

**Published:** 2021-06-17

**Authors:** X. Lan, S. Basu, S. Schwietzke, L. M. P. Bruhwiler, E. J. Dlugokencky, S. E. Michel, O. A. Sherwood, P. P. Tans, K. Thoning, G. Etiope, Q. Zhuang, L. Liu, Y. Oh, J. B. Miller, G. Pétron, B. H. Vaughn, M. Crippa

**Affiliations:** ^1^ Cooperative Institute for Research in Environmental Sciences University of Colorado Boulder Boulder CO USA; ^2^ Global Monitoring Laboratory National Oceanic and Atmospheric Administration Boulder CO USA; ^3^ Earth System Science Interdisciplinary Center University of Maryland College Park MD USA; ^4^ Global Modeling and Assimilation Office National Aeronautics and Space Administration Goddard Space Flight Center Greenbelt MD USA; ^5^ Environmental Defense Fund Berlin Germany; ^6^ Institute of Arctic and Alpine Research University of Colorado Boulder Boulder CO USA; ^7^ Department of Earth and Environmental Sciences Dalhousie University Halifax Nova Scotia Canada; ^8^ Istituto Nazionale di Geofisica e Vulcanologia Rome Italy; ^9^ Faculty of Environmental Science and Engineering Babes Bolyai University Cluj-Napoca Romania; ^10^ Department of Earth, Atmospheric, and Planetary Sciences Purdue University West Lafayette IN USA; ^11^ Joint Research Centre European Commission Ispra Italy

**Keywords:** atmospheric methane, atmospheric modeling, greenhouse gas, methane budget, source attribution, stable isotope of methane

## Abstract

We study the drivers behind the global atmospheric methane (CH_4_) increase observed after 2006. Candidate emission and sink scenarios are constructed based on proposed hypotheses in the literature. These scenarios are simulated in the TM5 tracer transport model for 1984–2016 to produce three‐dimensional fields of CH_4_ and *δ* ^13^C‐CH_4_, which are compared with observations to test the competing hypotheses in the literature in one common model framework. We find that the fossil fuel (FF) CH_4_ emission trend from the Emissions Database for Global Atmospheric Research 4.3.2 inventory does not agree with observed *δ* ^13^C‐CH_4_. Increased FF CH_4_ emissions are unlikely to be the dominant driver for the post‐2006 global CH_4_ increase despite the possibility for a small FF emission increase. We also find that a significant decrease in the abundance of hydroxyl radicals (OH) cannot explain the post‐2006 global CH_4_ increase since it does not track the observed decrease in global mean *δ* ^13^C‐CH_4_. Different CH_4_ sinks have different fractionation factors for *δ* ^13^C‐CH_4_, thus we can investigate the uncertainty introduced by the reaction of CH_4_ with tropospheric chlorine (Cl), a CH_4_ sink whose abundance, spatial distribution, and temporal changes remain uncertain. Our results show that including or excluding tropospheric Cl as a 13 Tg/year CH_4_ sink in our model changes the magnitude of estimated fossil emissions by ∼20%. We also found that by using different wetland emissions based on a static versus a dynamic wetland area map, the partitioning between FF and microbial sources differs by 20 Tg/year, ∼12% of estimated fossil emissions.

## Key Points

- Increased fossil fuel emissions are unlikely the dominant driver for post‐2006 global CH_4_ increase
- A significant decrease in the abundance of hydroxyl radicals (OH) cannot explain the post‐2006 global CH_4_ increase
- CH_4_ emission attributions are sensitive to the uncertainties in OH fractionation, tropospheric Cl sink, and wetland areas

## 1 Introduction

**Figure 1:**
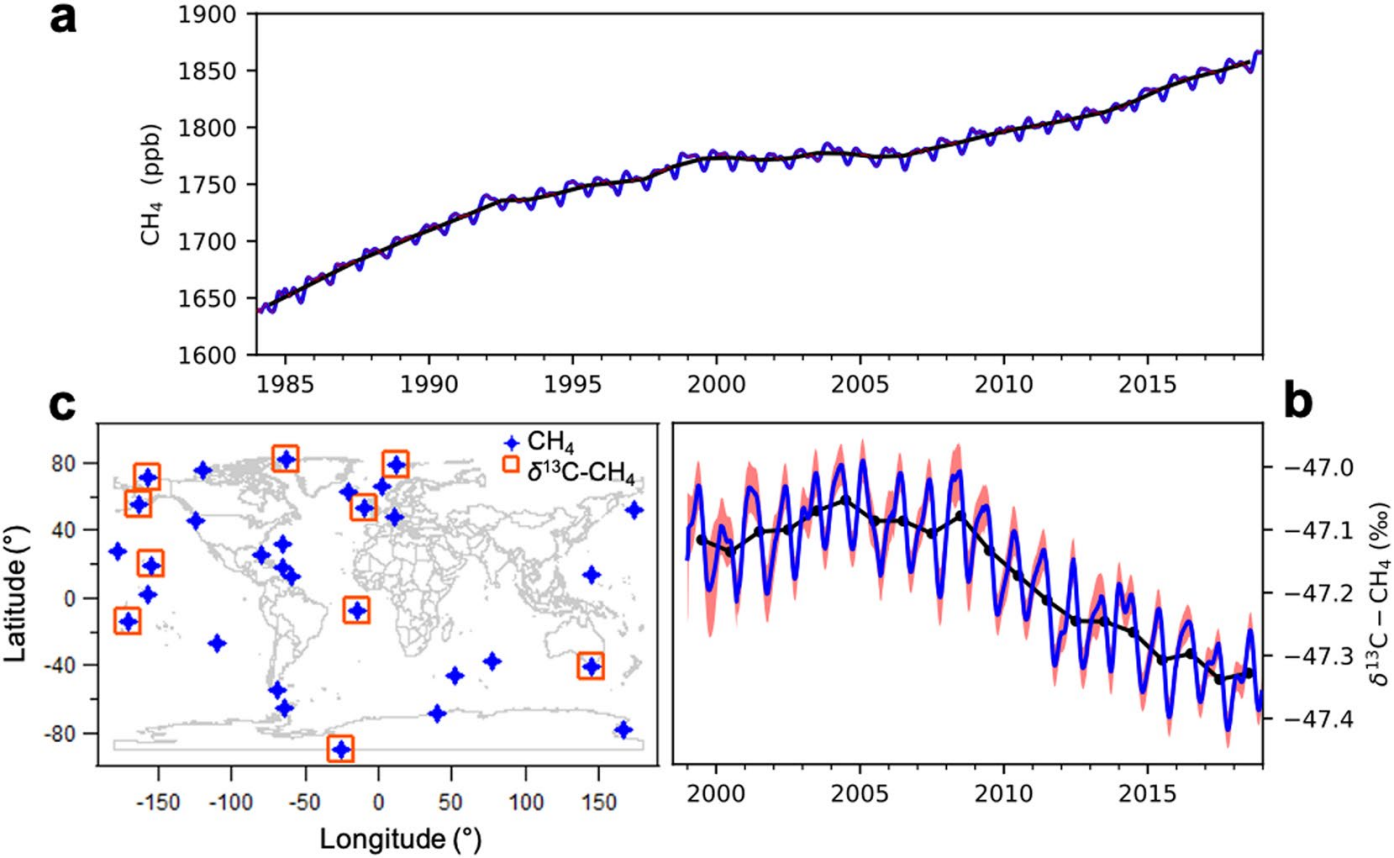
Globally averaged atmospheric CH_4_ (a) and *δ* ^13^C‐CH_4_ (b) from NOAA's Global Greenhouse Gas Reference Network; the blue curves in (a) and (b) are approximately weekly data and the red shaded areas are their uncertainty bounds (note uncertainties are too small to be visible in (a)), and the black curves are annual means. See Section 2.1 for uncertainty calculation. (c) The marine boundary layer sites from this network with CH_4_ and *δ* ^13^C‐CH_4_ measurements used in this study.

Atmospheric CH_4_ is the greenhouse gas responsible for the second largest increase in direct radiative forcing since 1750 (https://www.esrl.noaa.gov/gmd/aggi/; Forster et al., 2007). Globally distributed long‐term observations show that the atmospheric burden of CH_4_ has been increasing since 2007 after a relatively stable period from 1999 to 2006 (Figure 1). Around the same time that the increase started, the ratio of stable carbon isotopes of CH_4_ (^13^C/^12^C), denoted by *δ* ^13^C‐CH_4_, started to decrease after two centuries of increase (Ferretti et al., 2005; Michel et al., 2021) (Figure 1). Atmospheric CH_4_ abundance and its associated *δ* ^13^C‐CH_4_ result from the combined effect of emission and sink processes, including emissions from fossil sources, wetlands (WLs), rice, waste/landfills, ruminants, and biomass/biofuel burning (BB), and sinks from soil bacteria consumption, reactions with hydroxyl radicals (OH), chlorine radical (Cl), etc. (Saunois et al., 2020). Different CH_4_ sources have distinct *δ* ^13^C‐CH_4_ signatures over large spatial scales (Schwietzke et al., 2016) and different CH_4_ sinks have different preference for oxidation of ^12^C over ^13^C (Feilberg et al., 2005; King et al., 1989; Saueressig et al., 1995, 2001). Thus, high quality and representative measurements of both CH_4_ and *δ* ^13^C‐CH_4_ can provide independent constraints on CH_4_ emissions and sinks.

Emissions of CH_4_ can be estimated by top‐down and bottom‐up approaches. The top‐down approach relies on interpreting temporal and/or spatial differences in atmospheric measurements and a tracer transport model (e.g., CarbonTracker‐CH_4_, http://www.esrl.noaa.gov/gmd/ccgg/carbontracker-ch4/) or even a 1‐box model (e.g., Schwietzke et al., 2016). The bottom‐up approach is based on (i) production/economic statistics (e.g., fossil fuel [FF] emissions from an inventory are based on emission factors and FF‐related activities such as extraction, consumption, and distribution loss), (ii) scaling‐up flux measurements from local/regional scales studies to larger scales, and/or (iii) process‐based modeling. Even though the bottom‐up approach for extrapolation can be data driven, large uncertainty still exists given a limited amount of available data and the possibility that some emission processes may not be represented in the inventories and some emissions are double counted. The reported discrepancies in the global CH_4_ emission estimates for 2008–2017 between top‐down approaches (mean 576 Tg/year, range 550–594, Tg/year in this study refers to Tg or 10^12^ g of CH_4_) and bottom‐up approaches (mean 737 Tg/year, range 594–881) are significantly large, especially in WLs and other natural emissions (Saunois et al., 2020). However, it is difficult to distinguish CH_4_ emissions between natural and anthropogenic sources based on atmospheric CH_4_ data alone. The relatively smaller discrepancies in anthropogenic emissions may partially be due to the top‐down models' tendency to stay close to prior emission estimates from bottom‐up inventories given the sparse atmospheric data. Although this is understandable given that we generally have more statistics about human activities than natural processes, it can limit the influence of atmospheric data on optimizing anthropogenic emissions and further bias the estimates of natural emissions.

Large uncertainties also exist in CH_4_ sinks. Reaction with OH is the largest global sink of CH_4_. However, a direct measurement of the global OH abundance and distribution is not possible, thus the decay in atmospheric methyl chloroform (MCF) burden after its production was controlled by the Montreal Protocol are often used to estimate its atmospheric spatiotemporal variability, although with considerable uncertainty (Montzka et al., 2011; Rigby et al., 2013, 2017). The magnitude and distribution of the tropospheric chlorine (Cl) sink are also uncertain. A recent study based on chemical transport modeling proposed a significantly smaller tropospheric Cl sink of 13 Tg/year (Hossaini et al., 2016), with a different spatial distribution informed by the sources of tropospheric Cl (including the oxidation of anthropogenic and natural chlorocarbons and sea salt aerosol dechlorination), compared to a previous study examining the observed apparent ^13^C:^12^C kinetic isotope effect in the remote Southern Hemisphere (SH; 13–37 Tg/year, Allan et al., 2007). A study based on ^13^CO measurements as an indicator for isotopic composition of reacted CH_4_ suggested an even smaller role for the tropospheric chlorine sink (Gromov et al., 2018). The implications of these uncertainties on the global CH_4_ budget are further investigated here.

Given that the CH_4_ emissions and sinks are still grossly underconstrained by existing observations, many hypotheses have been proposed to explain the observed long‐term trends and variability of atmospheric CH_4_. Schaefer et al. (2016) proposed a dominant role for increased tropical agriculture emissions for the post‐2006 increase in global atmospheric CH_4_. Nisbet et al. (2016, 2019) suggested a stronger contribution from increasing tropical WL and agriculture emissions. Worden et al. (2017) proposed that a decrease in biomass burning accompanied by a moderate increase in FF emissions could explain the observed global CH_4_ trend. However, these studies mainly use CH_4_ and *δ* ^13^C‐CH_4_ data in box models that assume the global atmosphere is composed of one or a few boxes with homogenous emissions and losses in each box, and transport that connects the boxes (for multiple‐box models). Thus, they can be susceptible to biases caused by these simplified air transport and sink processes. Significant changes in CH_4_ sinks have also been proposed. A large decrease in global soil CH_4_ sink was found from long‐term measurements and data reviews (Ni & Groffman, 2018). Box model studies based on MCF suggested that a decrease in [OH] can explain the post‐2006 CH_4_ increase without sudden changes in CH_4_ emissions (Rigby et al., 2017; Turner et al., 2017); however, the [OH] trend estimated by using MCF in a box model may be biased, as shown by Naus et al. (2019) who use a global 3D transport model (TM5) to derive species‐ and time‐dependent quantities to drive 2‐box model simulations of MCF and CH_4_ to infer OH. In our study, we further evaluate these hypotheses using TM5 by constructing candidate emission and sink scenarios and running the model forward from 1984 to 2016 (see Section 2.4 for detail).

Different CH_4_ sources have distinct *δ* ^13^C‐CH_4_ signatures over large spatial scales (Schwietzke et al., 2016). The *δ* ^13^C‐CH_4_ signatures from sources are fully coupled with CH_4_ emissions, given that ^13^CH_4_ is a component of atmospheric CH_4_ itself. This is not the case for other coemitted but independent gas species, for example, C_2_H_6_ from FF emissions that are decoupled from CH_4_ emissions at large spatiotemporal scales (Lan et al., 2019). The *δ* ^13^C‐CH_4_ data and source signatures can provide strong additional constraints on CH_4_ emissions. However, models that use *δ* ^13^C‐CH_4_ as a constraint are sensitive to the assumed mean *δ* ^13^C‐CH_4_ source signatures. For example, changing the global average FF *δ* ^13^C‐CH_4_ signature from −39‰ to −44‰ based on an enlarged data set of *δ* ^13^C‐CH_4_ from fossil geochemistry data increased the estimate of global fossil emissions (FEs) by 50 Tg/year (Schwietzke et al., 2016). Thus, a key to accurately partition emissions to different source categories using atmospheric *δ* ^13^C‐CH_4_ observations is to apply *δ* ^13^C‐CH_4_ source signatures that accurately represent the study area. Before the large data set of *δ* ^13^C‐CH_4_ source signatures was available from Schwietzke et al. (2016), the source signatures used in previous global CH_4_ budget studies were either based on limited studies or not representative of global means (Schwietzke et al., 2016). Sherwood et al. (2017) further update the *δ* ^13^C‐CH_4_ signature data set over Schwietzke et al. (2016). Sherwood et al. (2017) also note a wide range of *δ* ^13^C‐CH_4_ values from each emission category, which is partially due to their spatial differences. Here, we continue the effort to update the data set (see Section 2.2).

Since spatial differences of atmospheric CH_4_ and *δ* ^13^C‐CH_4_ are apparent from the current measurement networks, we use spatially resolved *δ* ^13^C‐CH_4_ source signature maps developed in this and other studies in our model, which can further leverage the spatial information from atmospheric measurements and emission inventories to partition emissions at continental/regional scales. Our modeling approach will thus improve the constraint of *δ* ^13^C‐CH_4_ on the CH_4_ budget compared with box models, which are not designed to be used with detailed spatial information or previous 3D modeling studies with *δ* ^13^C‐CH_4_ that use one mean value globally for most source categories (e.g., Bousquet et al., 2006; Rice et al., 2016).

In this study, we aim to test the robustness of different hypotheses in reproducing observed long‐term trends, interannual variability, and spatial gradients of CH_4_ and *δ* ^13^C‐CH_4_. The roles of several CH_4_ sinks are also tested in model scenarios within the context of *δ* ^13^C‐CH_4_ mass balance, given that different sinks consume ^12^CH_4_ and ^13^CH_4_ at different rates.

## 2 Methods

### 2.1 Measurements and Marine Boundary Layer References

Observational data used to evaluate model results are from surface flask‐air measurements from NOAA's Global Greenhouse Gas Reference Network (Dlugokencky et al., 2019). Weekly air samples are collected in pairs of 2.5 L borosilicate glass flasks and sent to NOAA's Global Monitoring Laboratory in Boulder, Colorado, for CH_4_ analysis by gas chromatography with flame ionization detection. All CH_4_ data are reported on the WMO X2004A mole fraction scale (Dlugokencky et al., 2005) and reported in units of nmol mol^−1^ dry air and abbreviated “ppb” for parts per billion. Uncertainties are assigned to each measurement based on analytical repeatability and reproducibility, and uncertainty in propagating the X2004A standard scale to working standards (see SI for details).

A subset of the flask‐air samples is then analyzed for *δ* ^13^C‐CH_4_ at the Institute of Arctic and Alpine Research (INSTAAR), University of Colorado, Boulder. Gas chromatography‐Isotope‐ratio mass spectrometry is used for *δ* ^13^C‐CH_4_ analysis, and more details are in Miller et al. (2002). The *δ* ^13^C‐CH_4_ in air measurements are referenced against the Vienna Pee Dee Belemnite (VPDB) standard, with the definition *δ* ^13^C‐CH_4_ = (^12^C/^13^C)_sample_/((^12^C/^13^C)_VPDB_ − 1) × 10^3^ and reported in per mil (‰). Measurements of *δ* ^13^C‐CH_4_ are tied to the VPDB‐CO_2_ scale with methane‐in‐air standard gas. The INSTAAR realization of VPDB‐CO_2_ was established by calibration of INSTAAR whole air reference gases against the reference material NBS‐19 performed at the University of California Irvine (Tyler, 1986). Measurements of a surveillance cylinder throughout the measurement record validate the stability of the *δ* ^13^C‐CH_4_ scale. Observational data used in this study have also been through quality control and quality assurance including filters for samples with bad pair‐agreement, deficient peak height, standard drift, statistical outliers, and with other potential sampling and analysis errors.

A subset of the global network air sampling sites predominantly influenced by well‐mixed background air is used to construct zonal averaged surfaces using methods developed by Masarie and Tans (1995), to represent the observation‐based Marine Boundary Layer (MBL) global mean trend and latitudinal gradient. This includes 31 sites with CH_4_ measurements during the study period of 1984–2016 and 10 of which with *δ* ^13^C‐CH_4_ measurements starting in 1998 (Figure 1c). The observed global means of CH_4_ and *δ* ^13^C‐CH_4_ and their latitudinal gradients discussed in the following text refer to those from the MBL observations. Their uncertainties are estimated using nonparametric statistical methods that vary the network distribution and include analytical uncertainty (Dlugokencky et al., 1994). The combined uncertainties vary slightly with time but are typically less than 0.8 ppb (68% confidence interval) for global annual mean CH_4_. For global annual mean *δ* ^13^C‐CH_4_, uncertainties are estimated by accounting for analytical and atmospheric uncertainties, network distribution, and bias uncertainty (see SI for details). The combined uncertainties of global annual mean *δ* ^13^C‐CH_4_ since 2000 range from 0.016‰ to 0.028‰ (presented in Figure 5). More details on the MBL data products can be found at https://www.esrl.noaa.gov/gmd/ccgg/mbl/mbl.html. For model‐observation comparisons, model results from the same set of MBL sites are sampled, and the same calculation methods are applied to model results and observations for global mean and latitudinal gradient.

### 2.2 *δ* ^13^C‐CH_4_ Source Signatures

Gridded global maps of *δ* ^13^C‐CH_4_ source signatures were created largely based on our updated global source signature database, the Global *δ* ^13^C‐CH_4_ Source Signature Inventory 2020 (Sherwood et al., 2021), which was compiled using peer‐reviewed literature and conference and government reports.

Similar to the 2017 version (v2017) of the database (Sherwood et al., 2017), the new 2020 version (v2020, Sherwood et al., 2021) FF *δ* ^13^C‐CH_4_ data were categorized by (1) coal gas, (2) conventional gas, and (3) shale gas. The global gridded map of FF *δ* ^13^C‐CH_4_ signatures is created based on the spatial distribution of available *δ* ^13^C‐CH_4_ signatures. For most cases, country‐level mean signatures are used for all relevant emission grid cells for that country, separating Oil and Natural Gas (ONG) and coal sources. The country‐level mean ONG signatures were assumed to be time invariant over the study period; nevertheless, as the FF emissions (from Emissions Database for Global Atmospheric Research [EDGAR] 4.3.2 in our case) from individual countries who have different mean FF signatures changed over time, the global mean ONG *δ* ^13^C‐CH_4_ signatures also changed, and our model accounts for this impact while simulating atmospheric *δ* ^13^C‐CH_4_. However, for the U.S. where shale gas production is large with considerable variations from different basins in the past decades (according to available data from the U.S. Energy Information Agency: https://www.eia.gov/naturalgas/weekly/), the U.S. ONG signature is expected to change. That is because (i) there are temporal changes in basin‐level volumes of produced and possibly released conventional gas and shale gas that are associated with different signatures (see the v2020 database) that can change the basin mean ONG signature and (ii) ONG production and possibly their associated emission across different basins in the U.S. have also changed in the past decade. To address this point, we calculate the U.S. ONG *δ* ^13^C‐CH_4_ mean signature year by year as the average of shale gas and conventional gas signatures for major basins weighted by their respective basin‐level gas production volumes. We use production as a proxy for emission in calculating U.S. weighted mean signature because the magnitudes and temporal changes in U.S. basin‐level emissions were not well characterized. While similar temporal changes in the country‐level ONG *δ* ^13^C‐CH_4_ signature may also occur in other countries, often, little to no data are available, and thus country‐level temporal changes are only modeled for the U.S. U.S. shale gas production accounted for 87% of the global shale gas production in 2015 (EIA, International Energy Outlook 2016 and Annual Energy Outlook, https://www.eia.gov/todayinenergy/detail.php?id=27512). Thus, an improvement in representing U.S. shale gas in the global ONG signatures can largely reflect the overall impact from global shale gas development. For countries without available FF *δ* ^13^C‐CH_4_ signature data, global average *δ* ^13^C‐CH_4_ values (weighted by country‐level production) are used. This results in global coverage *δ* ^13^C‐CH_4_ signature maps for coal and ONG that can be used with various FF CH_4_ emission maps. Note that for coalbed CH_4_, we follow EDGAR and IPCC's classification and group it with emissions from coal production.

Biomass burning, biofuel burning, ruminant, and wild animal source CH_4_ emission signatures depend strongly on the locally available mix of C_3_‐ and C_4_‐based biomass material (for combustion or as a food source). Here, we used the averages of two different global maps of biomass C_3_/C_4_ ratios (Randerson et al., 2012; Still et al., 2003) in combination with measurements of C_3_‐ and C_4_‐based *δ* ^13^C‐CH_4_ source signatures to create global source signature maps at 1° × 1° resolution. For ruminants and wild animals, adjustments were applied to match the observed *δ* ^13^C‐CH_4_ signatures after accounting for the *δ* ^13^C‐CH_4_ changes during food processing, in addition to the distinction between C3‐ and C4‐feed in the diet itself. See SI for detailed approaches.

**Figure 2:**
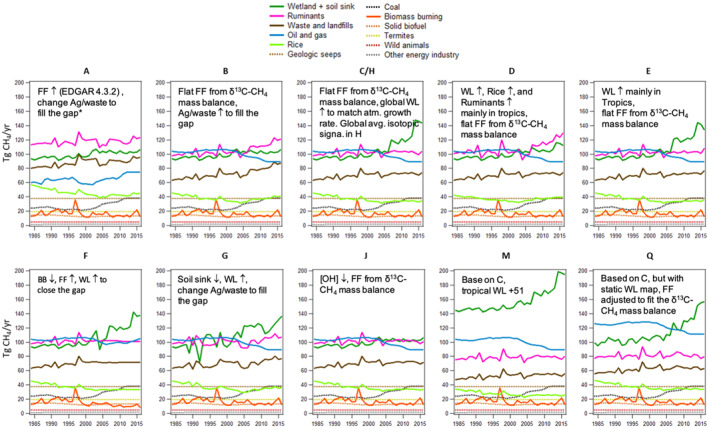
CH_4_ emission scenarios with hypothesis overview. *The “gap” refers to the differences between bottom‐up and top‐down emission estimates. The symbols “↑” and “↓” indicate positive and negative trends, respectively. See Section 2.4 for description of each scenario.

A spatially resolved global map of *δ* ^13^C‐CH_4_ signatures from geological seepage was developed by Etiope et al. (2019) and first used for atmospheric modeling in this study. For WL emissions, the spatial map of *δ* ^13^C‐CH_4_ from Ganesan et al. (2018) is used. We use globally averaged *δ* ^13^C‐CH_4_ source signatures for waste/landfills, termites, rice, and other energy/industry, given insufficient measurement sample size to develop spatial distributions. The other energy/industry category includes small FF sources (see Figure 2 for emission magnitude), such as power industry, combustion for manufacturing, aviation, ground transportation and shipping, and iron and steel production, and we use the global weighted average FF *δ* ^13^C‐CH_4_ source signature for this category.

### 2.3 Isotopic Mass Balance

Atmospheric CH_4_ sees the combined effects of all CH_4_ sources and sinks, thus the global mass balance of CH_4_ must be satisfied when we derive CH_4_ budgets for modeled scenarios. Considering the global atmosphere as one box with mass conservation, the mass balance of CH_4_ can be expressed on a yearly time scale (*t* = 1 year) as

(1) $$\frac{d\left({\text{CH}}_{4}\right)}{dt}=Q_{\text{Atm}}-\frac{\left[{\text{CH}}_{4}\right]}{\tau }\;$$

where [CH_4_] is the global burden and *τ* is the atmospheric lifetime. Equation 1 indicates the global annual atmospheric CH_4_ increase$\;\frac{d\left({\text{CH}}_{4}\right)}{dt}$ is caused by the imbalance between total emissions to the atmosphere $Q_{\text{Atm}}$, and total sinks expressed as $\frac{\left[{\text{CH}}_{4}\right]}{\tau }$. Total emissions to the atmosphere $Q_{\text{Atm}}$ includes subcategories of emissions (Q) from microbial (Mic), FE (including FF and natural geological seeps) and BB sources as

(2) $$Q_{\text{Atm}}=Q_{\text{Mic}}+Q_{\text{FE}}+Q_{\text{BB}}\;$$

A similar equation can also be written for $\delta^{13}C_{\text{atm}}$

(3) $$\delta^{13}C_{Q}\times Q_{\text{Atm}}=\delta^{13}C_{\text{Mic}}\times Q_{\text{Mic}}+\delta^{13}C_{\text{FE}}\times Q_{\text{FE}}+\delta^{13}C_{\text{BB}}\times Q_{\text{BB}}$$

where $\delta^{13}C_{x}\;$on the right‐hand side is the emission‐weighted source signature of a specific category of emissions. $\delta^{13}C_{Q}$ in the left‐hand side is the combined signal of *δ* ^13^C emitted to the atmosphere, which can be estimated using Equation 4 by assuming known CH_4_ sinks from our 3D tracer transport model (see details for modeled sinks in Section 2.5).

Atmospheric $\delta^{13}C_{\text{Atm}}$ also results from the combined effects of emissions and sinks on ^13^C/^12^C. Thus, the global mass balance of ^13^CH_4_ also needs to be satisfied when we derive CH_4_ budget. All sink processes enrich the atmosphere with ^13^C due to their faster reactions with ^12^C than ^13^C. Similar to the impact of different source signatures, different sink processes are distinguished by their different relative preference for oxidation of ^12^C over ^13^C, that is, the kinetic isotopic effect. The isotopic fractionation factor, *α*, is defined as the ratio of reaction rate constants for reactions with ^13^CH_4_ relative to that for ^12^CH_4_. The mass balance relationship for *δ* ^13^C‐CH_4_ is described in Equation 4, where *ε* is defined as the sink‐weighted average fractionation factor due to reactions with OH, Cl, and O(^1^D) and the soil sink, each with different fractionation. *ε* is commonly expressed as a negative value with ‰ unit, while *α* = 1 + *ε* is slightly smaller than 1. $\delta^{13}C_{\text{Atm}}$ and $\delta^{13}C_{Q}$ are linked through sink‐weighted fractionation:

(4) $$\delta^{13}C_{Q}=\alpha \times \delta^{13}C_{\text{Atm}}+\varepsilon \;$$

Note that Equation 4 is true only when the atmosphere is in CH_4_ and ^13^C/^12^C steady state, which is very unlikely in the past few decades when CH_4_ emissions and their partitioning among subcategories were changing. Equation 4 is presented here only to facilitate discussions in the following sections, but we do not assume steady state in the calculation of $\delta^{13}C_{Q}$. Instead, we use the exact equation describing the isotopic offset between sources and atmosphere (SI, Equation 5), while assuming known *α* and *ε* based on modeled sinks in TM5. In our study period, the steady state approximation yields about 0.3‰ difference in the estimated$\;\delta^{13}C_{Q}$.

### 2.4 Total Emissions and Emission Scenarios

Eleven candidate emission scenarios covering 1984–2016 were constructed for 3D model runs. As described in more detail below, each scenario, or set of scenarios, was designed to test the degree to which the diverging hypotheses of source/sink processes in the literature explain observed trends and spatial distribution of CH_4_ and *δ* ^13^C‐CH_4_. While previous studies have also used atmospheric observations to test individual hypotheses, this is the first study to comparatively test multiple hypotheses in one consistent model framework with full 3D modeling.

We first estimated top‐down annual global total emissions ($Q_{\text{Atm}}$) using Equation 1 with observation‐based global atmospheric CH_4_ annual increase,$\;\frac{d\left({\text{CH}}_{4}\right)}{dt}$, and abundance, [CH_4_], and modeled lifetime, *τ* (see the following section and SI for modeled sinks and lifetime). A conversion factor of 2.763 Tg CH_4_/ppb, based on atmospheric mass and CH_4_ sink distributions in TM5, is used to convert global mean interannual increase in ppb to Tg of CH_4_. The top‐down estimates show step increases in emissions in 2007 and 2014, which sum up to a 46 Tg/year increase in annual global emissions in 2016 compared with those during the 1999–2006 stable period.

Next, we address the fact that bottom‐up emission estimates (from process models and inventories, see details below) do not necessarily match the total emissions from the global CH_4_ mass balance derived in the previous step. For bottom‐up emissions, we use GFED 4.1s for biomass burning for 1997–2016 (Van Der Werf et al., 2017) and annual emissions from the Reanalysis of Tropospheric chemical composition project before 1997 (Schultz et al., 2008), and the EDGAR 4.3.2 inventory for other anthropogenic emissions for 1984–2016 (https://edgar.jrc.ec.europa.eu/overview.php?v=432_GHG ; Janssens‐Maenhout et al., 2019). For natural FE, we use gridded emission from Etiope et al. (2019). Emission estimates from wild animals and termites are adopted from Bergamaschi et al. (2007). WL emissions are the biggest natural source of atmospheric CH_4_ and its uncertainties are also among the largest. In our study, WL emissions and upland soil consumption are generated by a process‐based model, the Terrestrial Ecosystem Model (TEM; Liu et al., 2020; Zhuang et al., 2004). The TEM contains a thermal model including freeze–thaw processes, a sophisticated hydrological model for both upland and WL, and a CH_4_ biogeochemistry model that represents soil CH_4_ production, oxidation, and transport from soils to the atmosphere. Large uncertainties remain in WL areas, which are essential model inputs. These uncertainties are tested in this study by constructing two different sets of WL emission simulations based on a static WL area map (Matthews & Fung, 1987) and a dynamic inundation map from remote sensing based observations (Surface WAter Microwave Product Series [SWAMPS]; Schroeder et al., 2015) combined with the Global Lakes and Wetlands Dataset (GLWD, Lehner & Döll, 2004; Poulter et al., 2017). While the choice of meteorological data also has a significant impact in estimating WL emissions using a process‐based model (Liu et al., 2020), the same meteorological data from Climate Research Unit (CRU TS4.01, Harris et al., 2014) are used for both cases in TEM to estimate emissions. So differences in these two cases only reflect the differences in WL areas in our study. WL emissions based on dynamic inundation WL areas, which include a small increase from 134 to 144 Tg/year from 1999 to 2006 mean to 2016, are used for most emission scenarios. Modeled upland soil sink also includes a small increase from −33 to −38 Tg/year from 1999 to 2006 mean to 2016. An additional emission scenario, *Q_static_WL*, is constructed with WL emissions based on a static WL map (Matthews & Fung, 1987), which shows a significant increase in WL emissions from 140 to 193 Tg/year from 1999 to 2006 mean to 2016 (see Q in Figure 2). See Table S1 for more details on bottom‐up inventories and the spatiotemporal patterns of emissions.

The bottom‐up total emissions, as the sum of bottom‐up inventory emissions and net WL and soil sink emissions from the TEM process‐based model, do not show the step increases in 2007 and 2014 (Figure S2). More generally, we find a large discrepancy between annual total top‐down and bottom‐up emissions. To satisfy the global mass balance of CH_4_, all candidate emission scenarios are designed to have the same annual total emissions as the top‐down estimates from previous step that are based on observed global atmosphere CH_4_ and modeled lifetime *τ* (see following section and SI for modeled lifetime).

We partition the FE and Mic emissions ($Q_{\text{FE}}$ and $Q_{\text{Mic}}$) from the total top‐down emissions ($Q_{\text{Atm}}$) using Equations 2 and 3, by assuming known BB emissions ($Q_{\text{BB}}$) from inventory, following the approach in Schwietzke et al. (2016). Note that we also have an alternate scenario investigating the implication of a potential negative BB trend over the study time period. The combined signals of *δ* ^13^C‐CH_4_ from all emissions ($\delta^{13}C_{Q}$) are estimated using SI, Equation 5 (simplified form is presented as Equation 4) by assuming known sinks from TM5 model (i.e., *ε* is calculated from modeled sinks which are discussed in the following section) and using atmospheric measurements of *δ* ^13^C‐CH_4_ (i.e., $\delta^{13}C_{\text{atm}}$). Thus, only two unknowns, $Q_{\text{FE}}$ and $Q_{\text{Mic}}$, are solved for using Equations 2 and 3. Microbial emissions in this study refer to the sum of WL and Ag/waste (Ag for agricultural) emissions which include emissions from rice, ruminants, wild animals, termites, and waste/landfills sources.

Different emission scenarios are created by first scaling the spatially distributed bottom‐up inventories to the FE and Mic estimates from the isotopic mass balance calculation, with the exception of scenario A_FF+. In this step, all subcategory emissions within FE or Mic receive the same global annual scaling factor. Additional modifications are then made in the sources and/or sinks to represent the diverging hypotheses in the literature to determine which scenarios may explain the atmospheric observations. To adjust emissions from each subcategory and each time step, a single factor is applied to all model grids globally unless latitudinal ranges are specified.

The hypotheses addressed in this study include the followings:

(1) Long‐term source attribution based on global CH_4_ and *δ* ^13^C‐CH_4_ mass balance suggests an upward revision in the magnitude of FF emissions in all years compared to inventories (Schwietzke et al., 2016), represented by the following three scenarios:

*A_FF+*: ONG and coal emissions are from EDGAR 4.3.2, which show increasing FF emission since 2006; total FE increase is 28 Tg/year from 1999 to 2006 mean to 2016. Ag/waste emissions are adjusted to match top‐down total for annual emissions. Including the annual emission increase of 10 Tg/year for WL, the total annual emission increase for Mic in 2016 is about 25 Tg/year from 1999 to 2006 mean. The total increases in FE and Mic are partially offset by the small increase in soil sink (5 Tg/year) and the small decrease in BB emissions (2 Tg/year) from bottom‐up estimates.

*B_Mic+*: WL emissions have increased from 134 to 144 Tg/year from 1999 to 2006 mean to 2016. Constant total annual FF emissions of 167 Tg/year are obtained by scaling up EDGAR 4.3.2 ONG emissions, because 167 Tg/year FF emissions best match the global mass balance of both CH_4_ and *δ* ^13^C‐CH_4_ (described in Section 2.3) under our default sink setup in TM5 (see Section 2.5). Ag/waste emissions are adjusted to match top‐down total CH_4_ annual emissions. Thus, the combined increases in WL and Ag/waste emissions are responsible for post‐2006 global emission increase in this scenario.

*C_WL+*: Constant total annual FF emissions of 167 Tg/year are used as in B; assume increasing WL emissions are fully responsible for the global emission increase since 2007. Thus, the post‐2006 total emission increase, with interannual variability, is fully assigned to WL emissions (i.e., 46 Tg/year increase from 1999 to 2006 mean to 2016). Finally, Ag/waste BU emissions are adjusted slightly to match the top‐down total mass balance.

(2) Renewed growth of atmospheric CH_4_ after 2006 is due to increased microbial emissions in the tropics (Nisbet et al., 2016, 2019), represented by the following two scenarios:

*D_trop_Mic+*: WL, rice, and ruminant emissions, especially from the tropics (i.e., the same latitudinal increases as in Nisbet et al. [2019], see SI for more details), are assumed responsible for post‐2006 increase; constant FF emissions of 167 Tg/year are used.

*E_trop_WL+*: As in C, but the post‐2006 total emission increase is mainly assigned to tropical WL; constant FF emissions of 167 Tg/year are used. See SI for more details.

(3) Renewed growth of atmospheric CH_4_ after 2006 is due to moderate increases in FF emissions, which is consistent with the *δ* ^13^C‐CH_4_ constraint in this scenario because biomass burning emissions are assumed to have decreased (Worden et al., 2017), represented by scenario:

*F_BB−*: A 3.7 Tg/year total decrease in annual emissions of biomass burning (including interannual variability) from the 2001–2007 to 2008–2014 time periods is paired with a total 15.5 Tg/year increase in annual emissions of FF as in Worden et al. (2017). Biomass burning emissions are extrapolated to 2015 and 2016 to have the similar interannual variabilities as GFED4.1s for those years. Constant emissions of 167 Tg/year are assumed for FF before it increases in 2007. WL emissions are scaled to fit top‐down emission increases which yield 40 Tg/year increase in WL emissions from 1999 to 2006 mean to 2016. The small decrease (5 Tg/year) in soil sink from bottom‐up estimates is unchanged. Ag/waste emissions are adjusted so that they are not contributed to the post‐2006 increases.

(4) Renewed growth of atmospheric CH_4_ after 2006 is due to moderate increases in FF emissions and this is consistent with the *δ* ^13^C‐CH_4_ constraint because the soil sink decreased (Ni & Groffman, 2018), represented by scenario:

*G_soil−*: Adjust the total soil sink, including its negative trend and variability, to the estimate by Ni and Groffman (2018) who proposed a ∼77% decrease in soil sink from 1988 to 2015. Compared with the 1999–2006 mean, soil sink decreases by 19 Tg/year in 2016. Constant emissions of 167 Tg/year are assumed for FF. The small decrease (2 Tg/year) in BB from bottom‐up estimates is unchanged. The required emission increases to match the total top‐down emission are from the 10 Tg/year increase from bottom‐up WL estimates and 19 Tg/year increase in Ag/waste emissions. See SI for details.

(5) Globally uniform *δ* ^13^C‐CH_4_ source signatures reduce performance in source attribution of emissions compared to spatially resolved source signatures for each emission category (Feinberg et al., 2018; Ganesan et al., 2018), represented by scenario:

*H_mean_sig*: Spatial information in *δ* ^13^C‐CH_4_ source signatures is removed (i.e., only use globally emission‐weighted *δ* ^13^C‐CH_4_ mean signatures for each emission category). Emissions in this scenario are identical as scenario C_WL+.

(6) Renewed growth of atmospheric CH_4_ after 2006 is due to a negative trend in atmospheric [OH] (Rigby et al., 2017; Turner et al., 2017), represented by scenario:

*J_[OH]−*: Total emissions are kept the same as scenario C_WL+ before 2006, after which they are kept constant at the mean level for 2002–2006; [OH] in our model is adjusted uniformly to match observed atmospheric increases in CH_4_ (i.e., increase in atmospheric CH_4_ is assumed to be solely from changes in [OH]; see Equation 1 for the relationship among emissions, sinks, and global CH_4_ growth). This results in ∼8% total decrease in [OH] during the past decade (with interannual variability), which is comparable with hypothesized declines in the literature (Rigby et al., 2017; Turner et al., 2017).

(7) Shifting a considerable amount of Mic emissions to southern tropical WLs can better match atmospheric observations (Saunois et al., 2016; Schwietzke et al., 2016):

*M_more_trop_WL*: Based on scenario C_WL+, but 51 Tg/year CH_4_ emissions are added to WL areas 0°S–25°S for the whole study period; 50 Tg/year CH_4_ emissions are removed from Ag/waste uniformly from the globe (as was done in Schwietzke et al. [2016]).

Including scenario Q_static_WL described earlier, 11 emission scenarios are created. To better test different hypotheses, we match the above emission scenarios as closely to their proposed hypothesis as possible; however, a complete match is sometimes unattainable because of a lack of spatial information in box models used in these studies. A brief summary of emission in each scenario is presented in Figure 2. See SI for more details related to the design of emission scenarios.

### 2.5 Atmospheric Modeling of CH_4_ and *δ* ^13^C‐CH_4_

Atmospheric CH_4_ mole fractions and *δ* ^13^C‐CH_4_ isotopic ratios were simulated from January 1, 1984 to January 1, 2017 by combining the surface fluxes and their isotopic source signatures as surface conditions in the TM5 tracer transport model that was driven by ECMWF ERA Interim meteorology (Krol et al., 2005). TM5 was run globally at 6° × 4° over 25 vertical sigma‐pressure hybrid levels, for total CH_4_ and ^13^CH_4_. For each source type, ^13^CH_4_ flux was derived from the corresponding CH_4_ fluxes and source‐specific isotope source signatures.

Atmospheric CH_4_ has four loss terms in TM5. Three of them are CH_4_ destruction reactions in the atmosphere, namely destruction by OH and Cl in the troposphere and stratosphere, and destruction by O(^1^D) in the stratosphere. CH_4_ is also consumed by microbes in upland soils. A 3D monthly varying climatological OH field was constructed in the troposphere by scaling the OH field of Spivakovsky et al. (2000). Scaling factors used are specified below. In all sink scenarios, stratospheric Cl, OH, and O(^1^D) fields were constructed from a run of the ECHAM chemistry transport model (Jöckel et al., 2006). The OH, Cl, and O(^1^D) fields were all climatological with monthly variations. For each of these chemical sinks of CH_4_, fractionation factors for each of the reactions were applied to separately simulate the destruction of ^13^CH_4._ The reaction between CH_4_ and OH has two fractionation factors of −3.9‰ (Saueressig et al., 2001) and −5.4‰ in published literature (Cantrell et al., 1990), which we applied in different sink scenarios in this study to test its impact. For CH_4_‐only runs in some previous studies, the soil sink has typically been modeled as a negative flux at the surface. However, this sink fractionates between ^12^CH_4_ and ^13^CH_4_, which can be expressed as *k*_13_/*k*_12_ (*k* is the reaction rate constant) between the uptake rates of two isotopologs (King et al., 1989). Therefore, we modeled the soil sink as a first‐order destruction reaction affecting only the surface layer of TM5. See SI for more details.

To summarize, three different sink scenarios were constructed. (i) Our default sink setup, where we applied the tropospheric Cl sink of Hossaini et al. (2016) and the OH field from Spivakovsky et al. (2000) was scaled by 0.901. (ii) No tropospheric Cl sink of CH_4_, with the OH field from Spivakovsky et al. (2000) scaled by 0.9255 to ensure similar long‐term CH_4_ loss across all sink scenarios. (iii) Same as the default setup in (i), except for a fractionation factor of −5.4‰ for the CH_4_ + OH reaction (Cantrell et al., 1990) instead of the −3.9‰ in our default sink setup (Saueressig et al., 2001). The three different sink scenarios are combined with 11 different emissions scenarios (with some adjustments on fluxes described in Section 4) for a total of 33 different atmospheric simulations with TM5. All three sink scenarios yield the same CH_4_ lifetime. We calculated the CH_4_ lifetime (Figure S1) from the decay of a CH_4_ tracer with a realistic initial field in 1984 and no sources. Despite the climatological OH, Cl, and O(^1^D) fields, the modeled CH_4_ lifetime is not constant but shows a downward trend of ∼1%/decade due to changing covariances between interannually varying meteorology and a climatological OH distribution (see SI for details). This relatively small trend should have little impact on our modeling results.

Modeling ^13^CH_4_ in the atmosphere requires special care to spin up the model to a quasi‐steady state to avoid initial condition artifacts during the analysis period. There are three relevant time scales, (i) the interhemispheric exchange scale of ∼1 year to equilibrate CH_4_ mixing ratio gradients across the tropics, (ii) the stratosphere–troposphere exchange scale of ∼5 years to equilibrate the vertical profile of CH_4_ mole fractions, and (iii) the CH₄ lifetime of ∼9 years. All other time scales, such as the times required for the atmospheric *δ* ^13^C‐CH_4_ and its interhemispheric gradient to relax to new steady states once sources are changed, are determined by these three time scales (Tans, 1997). In principle, the time required for atmospheric *δ* ^13^C‐CH_4_ to reach steady state can be significantly longer than even the CH₄ lifetime, depending on the size of the atmospheric CH₄ burden and how far off the initial *δ* ^13^C‐CH_4_ is. In practice, since we start from atmospheric CH₄ and *δ* ^13^C‐CH_4_ fields based on observations, the time required to relax to steady state should not be more than a few CH₄ lifetimes. We spun up our model for 16 years from 1984 to 1999 and selected 2000–2016 as our analysis period for *δ* ^13^C‐CH_4_. While a longer spin‐up period would have been better, we were limited by the fact that the further back we went in time the more uncertain and likely erroneous the initial CH₄ and *δ* ^13^C‐CH_4_ fields were going to be, offsetting the benefit of a longer spin‐up. Details on the initial CH_4_ and *δ* ^13^C‐CH_4_ fields are described in the SI.

## 3 Results

### 3.1 Updated *δ* ^13^C‐CH_4_ Source Signatures

**Figure 3:**
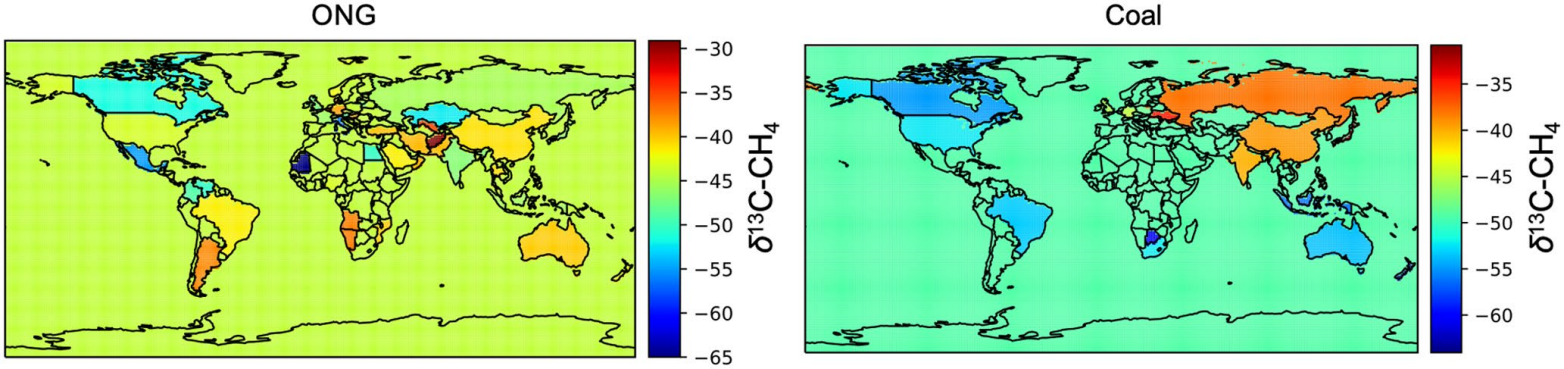
Country‐level *δ* ^13^C‐CH_4_ source signatures for ONG (2010) and coal emissions (assume time invariant). For grid cells without data, a global flux weighted mean is used. ONG, Oil and Natural Gas.

Compared with the v2017 of the source signature data set (Sherwood et al., 2017), the sample size for FF *δ* ^13^C‐CH_4_ signatures in v2020 is 8% larger (new total sample count is 9,477). The updated v2020 of *δ* ^13^C_FF_ samples is representative of FF emissions from 47 countries accounting for ∼81% of global ONG and ∼90% of global coal production. Figure 3 shows maps of ONG and coal *δ* ^13^C‐CH_4_ signatures. The inclusion of additional data has negligible effect on global mean FF signature, but there are regional differences in *δ* ^13^C_FF_ signatures when comparing with v2017. By accounting for the rapid development of shale gas production in the U.S. and the shifting ONG production across different basins, we find that the U.S. shale gas signatures have become heavier than conventional gas signatures (Figure S3; Milkov et al., 2020). This is in disagreement with Howarth (2019) who used a more depleted *δ* ^13^C‐CH_4_ signature for shale gas to support the hypothesis that the increase in U.S. FF emissions is the dominant contributor to the post‐2006 global CH_4_ increase. Given the shift toward more shale gas production relative to conventional gas, the U.S. ONG signature (as a production‐weighted mean of shale and conventional gas production) increased by 2.7‰ from 2006 to 2016.

For the Mic and BB signatures, the source signature database update results in a 74% increase of Mic samples (new total sample count is 1,776) and 3% increase of BB data (new total sample count is 935). The new gridded data with a larger sample size constitute a stronger constraint to attribute emissions to specific regions and individual source categories in modeling.

When we apply the default sink scenario and sink fractionation (*ε* = −7.85‰, in Equation 4) from TM5 (see default sink description in Section 2.5) in the mass balance equations, we estimate that 167 Tg/year FE (assuming no temporal trend) and 360–420 Tg/year Mic emissions (from 2000 to 2016) best matches the top‐down emission constraint combined with global mean signatures that are calculated by weighting grid‐level signatures and emissions. If we use *ε* = −6.3‰ as the total sink fractionation factor, the same as in Schwietzke et al. (2016), the FE constrained by the new grid‐level signature and emission is 210 Tg/year which is comparable with the 195 ± 32 Tg/year estimates for 2003–2013 from Schwietzke et al. (2016). This FE magnitude also in good agreement with the annual FE estimate of 168 ± 13 Tg/year for 1984–2000 based on radiocarbon (^14^CH_4_) measurements, when using −6‰ ± 2‰ as the total sink fractionation factor in their study (Lassey et al., 2007). A recent study (Fujita et al., 2020) found that the optimized emissions from CH_4_ inversion still underestimate FE and overestimate Mic emissions. FE was adjusted to 162 ± 2 Tg/year to best fit both *δ* ^13^C‐CH_4_ and *δ*D‐CH_4_ observations at Arctic and Antarctic surface stations.

### 3.2 Simulated Global Mean CH_4_ and Its Latitude Gradient

**Figure 4:**
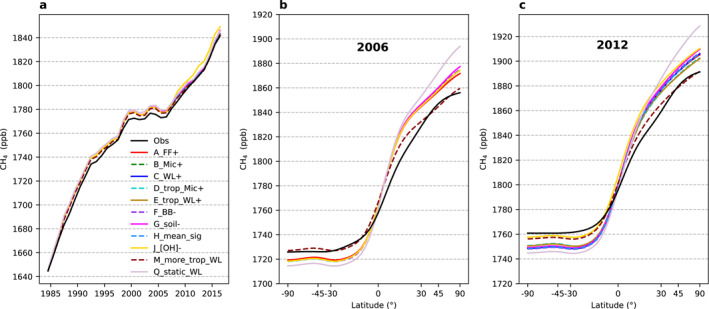
Modeled global mean CH_4_ (a) and annual mean latitudinal gradients ((b) for 2006 and (c) for 2012) from different emission scenarios, compared with those from Marine Boundary Layer observations (black). All scenarios show similar performances on global mean CH_4_ in (a) since they are constructed to be consistent with the atmospheric CH_4_ global mean growth rates.

Emission scenarios described in Section 2.4 are simulated in the TM5 transport model to produce 4D fields of atmospheric CH_4_ and *δ* ^13^C‐CH_4_. Simulated global mean CH_4_ of all scenarios compares reasonably well with observations (Figure 4a), which is expected since all scenarios were constructed to have global total emissions consistent with the atmospheric CH_4_ global mean growth rate. However, the agreements with the observations were not exact because the modeled chemical loss of CH_4_ depends on the amount of CH_4_ in each grid cell and therefore on the emission patterns. The lifetime calculated from a background CH_4_ tracer with no sources will be close but not be identical to the lifetime from a CH_4_ tracer with a specific emission pattern.

The comparison between modeled and observed MBL latitudinal gradients can provide information on the scenario‐based latitudinal distribution of emissions, assuming modeled interhemispheric transport is reasonably accurate. The accuracy of TM5's interhemispheric transport is evident from comparisons to the observed SF_6_ gradient at background sites (Basu et al., 2016). We use 2006 and 2012 as examples in Figures 4b and 4c since we find only small interannual variability in the observed annual mean latitudinal gradient after 1992. We find larger north‐to‐south gradients in most model scenarios compared to observations, with overestimates in the Northern Hemisphere (NH) and underestimates in the SH. These suggest that bottom‐up inventories have placed too much emission in northern latitudes and too little in low or southern latitudes. A steeper N‐S CH_4_ gradient in the model can, in principle, also arise from a ratio of OH in the NH to SH that is too low. However, the NH:SH OH ratio is 0.99 for Spivakovsky et al. (2000), and ratios significantly larger than 1 are not supported by observed MCF latitudinal gradients (Patra et al., 2014). Of all our scenarios, scenario M_more_trop_WL, which has more southern tropical emissions (51 Tg/year more in WL), yields by far the best match with observed latitudinal gradients.

### 3.3 Simulated Global Mean *δ* ^13^C‐CH_4_ and Its Latitude Gradient

**Figure 5:**
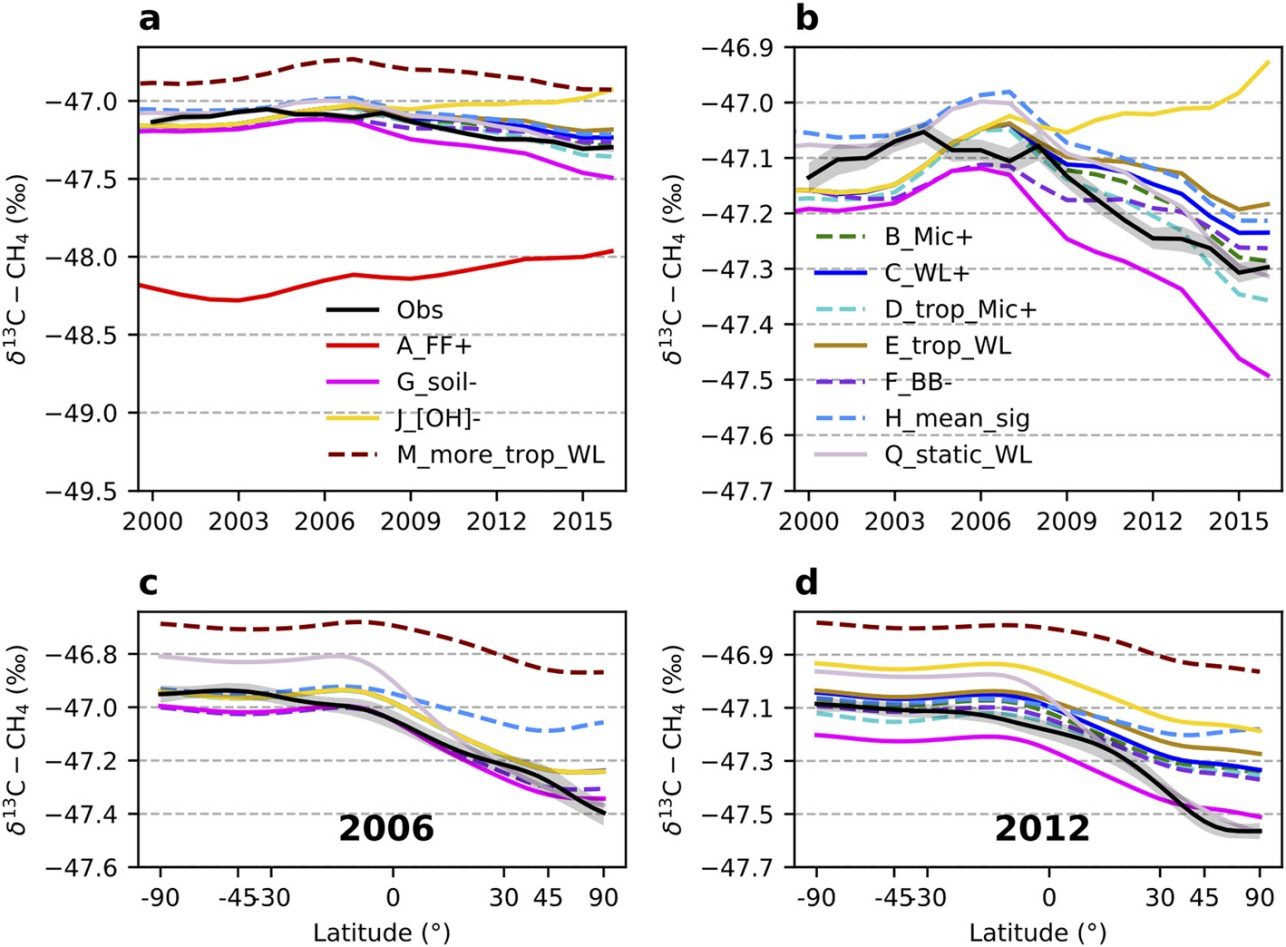
Modeled global mean *δ* ^13^C‐CH_4_ (a, b) and their latitude gradients (c, d) from different emission scenarios compared with those from Marine Boundary Layer observations. (b) A zoom‐in view of (a). The shaded area around the observations in (b)–(d) is estimated uncertainty bounds. See Section 2.1 for uncertainty calculation.

While simulated global mean CH_4_ generally compares well with observations that is not always the case for global mean *δ* ^13^C‐CH_4_. When using ONG and coal emissions from EDGAR 4.3.2, which show a generally positive trend from 1984 to 2016 and contribute to a total FE (including FF and geological seep emissions) increase from 120 to 150 Tg/year (Figure 2, scenario A_FF+), modeled *δ* ^13^C‐CH_4_ is significantly depleted and accompanied by a positive long‐term trend that contradicts the observed *δ* ^13^C‐CH_4_ decrease after 2008 (Figure 5a). The observed global *δ* ^13^C‐CH_4_ decrease is estimated to be ∼0.25‰ since 2008, which is a robust signal since its much larger than the global annual mean *δ* ^13^C‐CH_4_ uncertainties ranging from 0.016‰ to 0.028‰ (Figure 5b). The *δ* ^13^C‐CH_4_ modeled with scenario A_FF+ is ∼1‰ lower than observations. This is a result of the imbalance between emission and sink effects on *δ* ^13^C‐CH_4_, that is, the isotopic mass balance described in Section 2.3 is not satisfied in this scenario. To correct for this discrepancy, the magnitude of FF emissions needs an upward revision, if BB emissions, an isotopically heavier source, are still in line with inventories. Including ∼37 Tg/year emissions from geological seeps, we estimate that 167 Tg/year FE, that is, FF emissions account for 130 Tg/year, best match the isotopic mass balance (uncertainty from geological seeps emissions is discussed in Section 4.4). This value is used in 9 out of 11 emission scenarios (with scenario A_FF+ and part of scenario F_BB− as exceptions; see Section 2.4 for details), which results in reasonable agreements with the observed atmospheric *δ* ^13^C‐CH_4_ levels during CH_4_ stabilization period before 2007 (Figures 5a and 5b). The positive long‐term trend in modeled *δ* ^13^C‐CH_4_ from scenario A_FF+ that contradicts the observed decrease suggests that the increase in FF emissions of this size is unlikely, despite an equally large increase in Mic emissions in this scenario. Compared with 1999–2006 means, scenario A_FF+ has 25 Tg/year increase in Mic emissions and 28 Tg/year increase in FE emissions at 2016, accompanied by a small decrease in BB emissions. The FF emission magnitudes and the proportional increases of Mic and FF emissions in scenario A_FF+ are similar to the new top‐down estimates from the Global Methane Budget which averaged 92 Tg/year (range 70–113) FF emissions for 2000–2006 and concluded that agricultural and FF emission increases contribute equally to the post‐2006 global total emission increase (Jackson et al., 2020; Saunois et al., 2020). However, we do not expect these estimates to be consistent with atmospheric *δ* ^13^C‐CH_4_ observations because *δ* ^13^C‐CH_4_ observations and *δ* ^13^C‐CH_4_ source signatures were not used to constrain CH_4_ budget in the model studies used by the Global Methane Budget.

For the scenarios that generally agree well with the observed atmospheric *δ* ^13^C‐CH_4_ levels during CH_4_ stabilization period before 2007, we further compare different changes in their category emissions afterward as to whether they can track the recent negative trend in atmospheric *δ* ^13^C‐CH_4_. In seven scenarios (B–E, H, M, and Q), we assume constant total FF emission from 1984 to 2016 (while compensating trends exist for ONG and coal) and attribute all increase in global emissions since 2006 to Mic sources. Since *δ* ^13^C‐CH_4_ signatures from Mic emissions are generally more depleted than $\delta^{13}C_{Q}$ (see Fig. S4 and Equation 4), simulated global mean *δ* ^13^C‐CH_4_ decreases, consistent with observations, while Mic emissions increase (Figure 5b). When we attribute the increases in global emissions (∼46 Tg/year since 2006) mainly to increases in Ag/waste with only an 10 Tg/year increase in WL (scenario B_Mic+), we find slightly better agreement with the observed *δ* ^13^C‐CH_4_ decrease (Figure 5b) than by attributing all increases only to WL emissions (scenario C_WL+). This is because the *δ* ^13^C‐CH_4_ signatures from aggregated Ag/waste emissions are generally more depleted than from WL, with the exceptions of Arctic WL (see further discussions below). That is also the case when the increases are mainly in the tropics (scenarios D_trop_Mic+ and E_trop_WL+). However, when comparing the simulated latitudinal gradients with observations (Figures 5c and 5d, year 2006 and 2012 are used as examples), we cannot distinguish scenarios B_Mic+/C_WL+ from scenarios D_trop_Mic+/E_trop_WL+, which is likely because the latitudinal gradient generally reflects the average latitudinal distribution of emissions, while the influence of relatively smaller changes added to the averages is not apparent.

To better match the simulated and observed latitudinal gradients of CH_4_, we increase WL emissions from the southern tropics in scenario M_more_trop_WL, because large uncertainty remains in tropical WL emissions, while spatial distributions of anthropogenic emissions are more or less constrained by population metrics and human activity data in bottom‐up inventories. As shown in Figures 5c and 5d, this scenario overestimates global mean *δ* ^13^C‐CH_4_ and underestimates its N‐S difference. These are partially due to the heavier WL *δ* ^13^C‐CH_4_ signatures in the southern tropics (−53‰ to −58‰ between 0°S and 25°S) than high northern latitudes (<−65‰ for regions north of 50°N) (Ganesan et al., 2018). More southern tropical WL emissions in this scenario enrich the global mean WL source signatures. By increasing emissions in the southern tropics, we also remove Mic emissions from all Ag/waste to balance the global CH_4_ budget. But the global mean *δ* ^13^C‐CH_4_ signature of ruminants, the second largest Mic source after WL, is more depleted (∼−66‰, Figure S4) than the WL signatures in the southern tropics. Most scenarios overestimate CH_4_ in NH and underestimate in the SH, while underestimate the N‐S gradients for *δ* ^13^C‐CH_4_. These discrepancies then suggest a need to shift emissions from the NH midlatitudes to the tropics or SH while this change should increase the overall N‐S difference of *δ* ^13^C‐CH_4_. A plausible solution is to add more NH Mic emissions while decreasing FF or BB emissions in the NH, and/or shift NH FF or BB emissions to the tropics and SH. One example is scenario Q_static_WL that has more WL emissions from high northern latitudes (see Section 4.3 for more details); the N‐S difference of *δ* ^13^C‐CH_4_ in this scenario is enlarged. But Q overestimates the N‐S differences for both CH_4_ and *δ* ^13^C‐CH_4_. These call for simultaneous assimilation of both CH_4_ and *δ* ^13^C‐CH_4_ in modeling studies.

When a large positive trend in Mic emissions is not present, changes in other source or sink processes that can reduce atmospheric *δ* ^13^C‐CH_4_ are required to follow the observed negative trend in *δ* ^13^C‐CH_4_, for example, decreasing BB emissions in scenario F_BB− or a decreasing soil sink in scenario G_soil−. Scenario F_BB− with 3.7 Tg/year total decrease in annual BB emissions from the 2001–2007 to 2008–2014 time periods (a steeper decrease than the GFED 4.1s inventory) and 15.5 Tg/year total increase in FF emissions between the same periods, as proposed by Worden et al. (2017), gives a global *δ* ^13^C‐CH_4_ decrease of 0.11‰ since 2008, which is smaller than the observed decrease of 0.25‰. Based on the isotopic mass balance, a smaller increase than 15.5 Tg/year in FF emissions accompanied by a larger increase in Mic emissions can improve the fit to observations in this scenario. Even with the 15.5 Tg/year increase in FF emission (based on 167 Tg/year total FE magnitude) in current scenario, increase in FF emission is still not the dominant driver for the post‐2006 global CH_4_ increase because the Mic emission increase is larger in this scenario to meet the ∼46 Tg/year increase in global total emissions since 2006.

A significant negative trend in soil sinks, as proposed by Ni and Groffman (2018), accompanied by a small increase in WL emissions (i.e., the same WL emissions as the TEM process‐based model without additional increases) yields a global *δ* ^13^C‐CH_4_ decrease of 0.30‰ (scenario G_soil−) that is slightly larger than observed. Although soil sinks contribute to a small amount of total CH_4_ sinks (∼5%), this result illustrates how its large fractionation factor (−21‰, King et al., 1989) plays a significant role in observed *δ* ^13^C‐CH_4_.

Interestingly, a scenario (H_mean_sig) with source signatures that can well represent the global means but without spatial representation can already track the global decreases in *δ* ^13^C‐CH_4_, but the modeled *δ* ^13^C‐CH_4_ latitude gradient is smaller than those from a majority of emission scenarios and the observations. Thus, we can expect that the spatially resolved *δ* ^13^C‐CH_4_ source signatures will improve the spatial attributions of CH_4_ emissions. This confirms conclusions of Feinberg et al. (2018) and Ganesan et al. (2018).

The model scenario with a significant negative trend (−8%) in [OH] yields a positive trend in global mean *δ* ^13^C‐CH_4_ (scenario J_[OH]−), contradicting the observed decrease. Unlike Rigby et al. (2017) and Turner et al. (2017), which are based on box models with simplified CH_4_ sinks, we cannot match the global *δ* ^13^C‐CH_4_ trend by decreasing [OH] in our 3D model that specifically simulates individual sink processes. Different CH_4_ sinks fractionate differently between ^12^C and ^13^C. We find that the positive trend in simulated *δ* ^13^C‐CH_4_ from this scenario is mainly caused by the increase in the total sink‐weighted fractionation, which is discussed further in Section 4.

Note that in this study, we do not attempt to optimize emissions and their spatial distributions to best match observations. This will be done in a future inverse modeling study. Instead, we explore the potential for different emission and sink scenarios to match the large spatiotemporal patterns of MBL CH_4_ and *δ* ^13^C‐CH_4_, which can help us understand the leverage of different emission and sink scenarios in changing the total global budget and further improve the inverse modeling.

## 4 Discussion

### 4.1 Sensitivity of CH_4_ Budget Estimates to Tropospheric Cl and OH Fractionation

**Figure 6:**
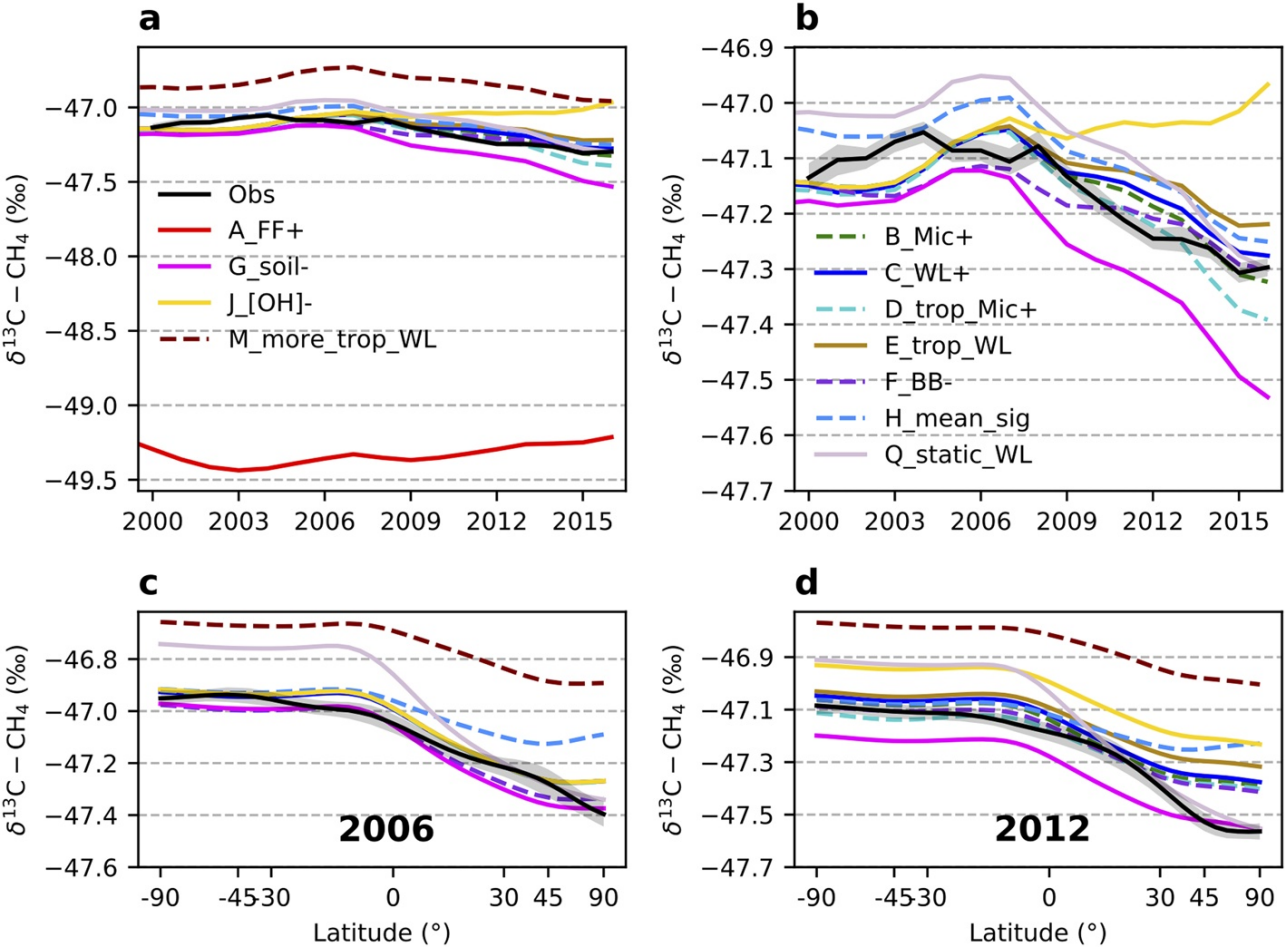
Modeled global mean *δ* ^13^C‐CH_4_ (a, b) and annual mean latitudinal gradients (c, d) from different emission scenarios combined with a sink scenario excluding tropospheric Cl. (b) A zoom‐in view of (a). The shaded area around the observations in (b)–(d) is estimated uncertainty bounds. See Section 2.1 for uncertainty calculation.

When investigating the partitioning of CH_4_ emissions using atmospheric *δ* ^13^C‐CH_4_ and source isotopic signatures, we need to make assumptions about the CH_4_ sinks as well. The default sink scenario in the above model runs includes ∼13 Tg/year CH_4_ sink from tropospheric chlorine atoms (Hossaini et al., 2016). Although this accounts for less than 3% of the total CH_4_ sink, the large fractionation of about −62‰ (Saueressig et al., 1995) makes tropospheric Cl potentially important for the atmospheric *δ* ^13^C‐CH_4_ budget. However, the concentration and distribution of tropospheric Cl are uncertain; we create an additional sink scenario that excludes tropospheric Cl and combine it with all emission scenarios to evaluate the sensitivity of the Cl sink to the partitioning of emissions. We fix total atmospheric emissions as in the previous emission scenarios and increase the OH sink to keep the total CH_4_ destruction the same as in the default sink scenario. From a mass balance point of view, atmospheric *δ* ^13^C‐CH_4_ responds to the sink‐weighted fractionations (*ε*) of the OH, Cl, stratospheric O(^1^D), and soil sinks. Removing a sink with a large fractionation effect results in a smaller sink‐weighted fractionation (*ε* changes from −7.85‰ in default sink scenario to −6.58‰). Changes in emission partitioning are required to compensate for this effect. We modify all emission scenarios except scenario A_FF+ for the ONG emissions in FE and ruminant emissions in Mic only, that is, one isotopically heavier and one lighter source, while keeping BB emissions unchanged. Otherwise, there are too many possible combinations of emission changes to investigate. To satisfy the mass balances of both CH_4_ and *δ* ^13^C‐CH_4_, we find that total annual FE has to increase to 200 Tg/year from 167 Tg/year while annual ruminant emissions decrease by the same amount. In these cases, modeled global mean *δ* ^13^C‐CH_4_ and latitudinal gradients from TM5 (Figure 6) are very similar to our previous model results with the default sink scenario in Figure 5, with exception of scenario A_FF+, which still does not satisfy the global mass balance of *δ* ^13^C‐CH_4_.

The impact of tropospheric Cl on *δ* ^13^C‐CH_4_ seasonal cycles in the extra tropics of Southern Hemisphere (ETSH) was explored by Allan et al. (2001). Based on the *δ* ^13^C‐CH_4_ seasonal cycles, they suggested a significantly larger fractionation for CH_4_ loss than was expected from the OH sink alone. Furthermore, Lassey et al. (2011) suggested that when there is a tropospheric Cl sink in ETSH the seasonal cycle amplitude of *δ* ^13^C‐CH_4_ is 3 times larger than those with only OH sink. However, our model results find no significant difference in *δ* ^13^C‐CH_4_ seasonal cycle amplitudes at Cape Grim, Australia (an ETSH site, 40.68°S) in sink scenarios with and without tropospheric Cl (Figure S5). Although we also include soil sinks and stratospheric sinks in both cases, our model results suggest that tropospheric Cl is not necessary to explain the observed *δ* ^13^C‐CH_4_ seasonal cycle amplitudes. Lassey et al. (2011) used nominal source and sink scenarios to simulate *δ* ^13^C‐CH_4_ seasonal cycles, which may not directly translate to a 4D model realization of the atmosphere from a more realistic source and sink setup. Removing tropospheric Cl as a CH_4_ sink (while OH sink was increased by the same amount) yields a 33 Tg/year change in estimated FE and Mic emission partitioning, which is within the uncertainty ranges proposed by previous studies (Saunois et al., 2020; Schwietzke et al., 2016). A recent study by Strode et al. (2020) has investigated the sensitivity of the atmospheric *δ* ^13^C‐CH_4_ to intermodel diversity in tropospheric Cl using a series of sensitivity studies with a global 3D model, while keeping emissions unchanged in the comparison. They found that the range of Cl field available from current global models leads to a wide range of simulated *δ* ^13^C‐CH_4_ values, and each percent increase in the amount of CH_4_ loss from Cl reaction increases global mean *δ* ^13^C‐CH_4_ by ∼0.5‰. It is expected from isotopic mass balance (described in Section 2.3) that increasing the amount of CH_4_ loss from Cl reaction can enrich atmospheric *δ* ^13^C‐CH_4_. Here, we demonstrate that changing FF and Mic emission partitioning in a reasonable range can still fit in the constrains of atmospheric CH_4_ and *δ* ^13^C‐CH_4_ observation. Thus, we cannot rule out the existence of a significant tropospheric Cl sink of ∼13 Tg/year as suggested by Hossaini et al. (2016). Future studies are required to better quantify the tropospheric Cl sink and its spatiotemporal variations given its importance in interpreting *δ* ^13^C‐CH_4_ signals.

**Figure 7:**
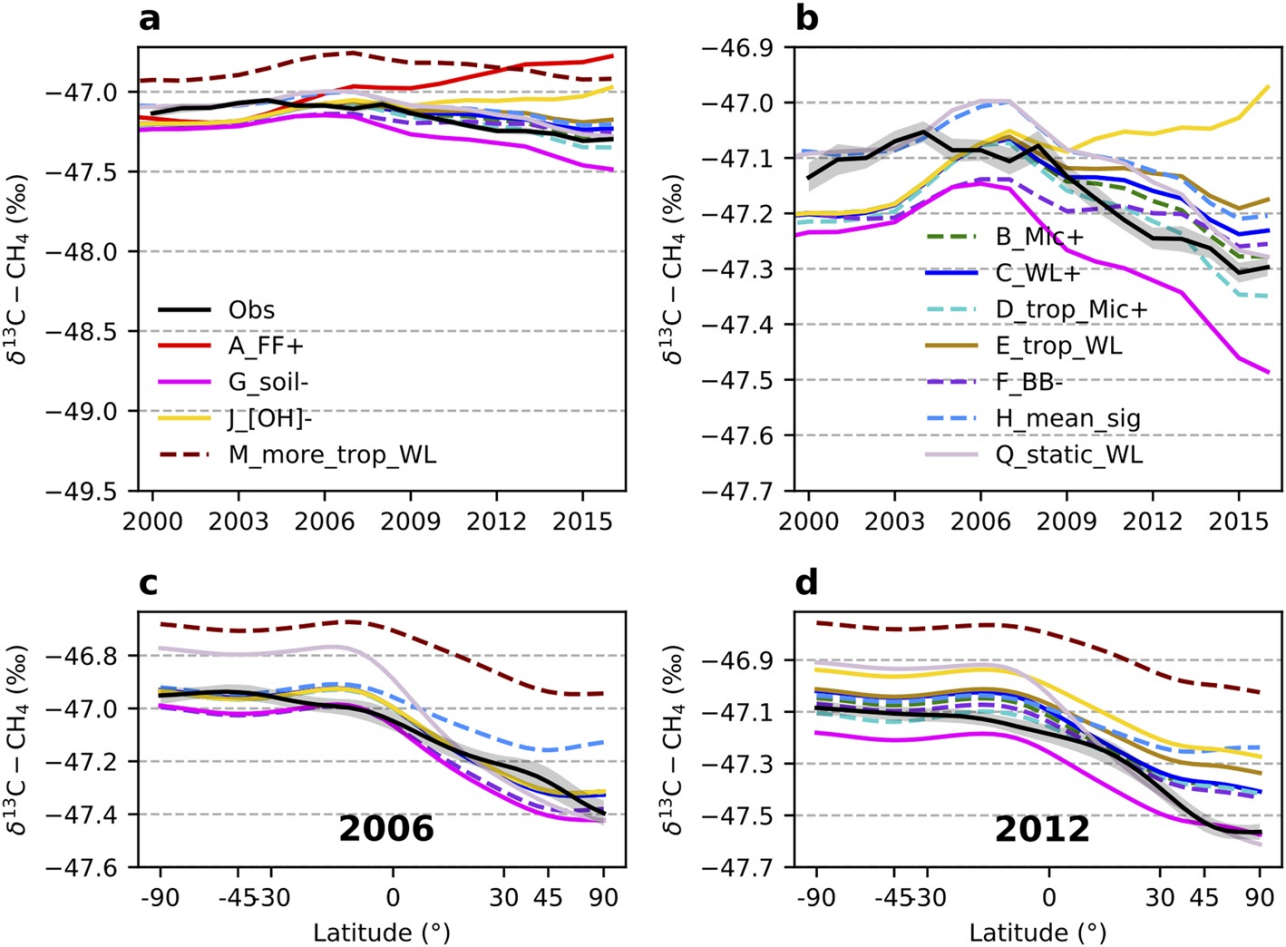
Modeled global mean *δ* ^13^C‐CH_4_ (a, b) and annual mean latitudinal gradients (c, d) from different emission scenarios combined with a sink scenario using OH fractionation of −5.4‰. (b) A zoom‐in view of (a). The shaded area around the observations in (b)–(d) is estimated uncertainty bounds. See Section 2.1 for uncertainty calculation.

Another source of uncertainty associated with CH_4_ sinks lies in the quantification of fractionation by OH (*ε*_OH_). An *ε*_OH_ of −3.9‰ (Saueressig et al., 2001) is used in our default sink scenario and the modified sink scenario without tropospheric Cl; but an *ε*_OH_ of −5.4‰ was reported by Cantrell et al. (1990). Since we cannot determine the relative merits of the reported OH fractionation, we evaluate both for a better understanding of the OH fractionation uncertainty and its impact on emission partitioning in previous literature and for future reference. Changing the *ε*_OH_ does not require modification of the CH_4_ sinks, but it changes the sink‐weighted *ε* from −7.85‰ to −9.03‰ (including tropospheric Cl in both cases). By modifying ONG and ruminant emissions in all emission scenarios but A to adapt to the new *ε*, we find that 135 Tg/year FE can best fit the new isotopic mass balance with −5.4‰ as *ε*_OH_. The modeled global mean *δ* ^13^C‐CH_4_ and latitude gradients from TM5 (Figure 7) are also similar to our previous model runs in Figure 5, with exception of scenario A_FF+.

These sensitivity tests suggest that different plausible sink scenarios require different emission partitioning to reproduce observation‐based global mean CH_4_ and *δ* ^13^C‐CH_4_ and their large‐scale distributions. For the two modified sink scenarios explored here, we found that transferring a significant amount of CH_4_ emissions (>30 Tg/year) from FF emissions to ruminant emissions led to similar level of agreements as the default scenarios. For another potential sink scenario with −5.4‰ as *ε*_OH_ but without tropospheric Cl, our calculations show the same sink‐weighted *ε* as in the default sink scenario; the partition of emissions among sources is thus the same. While many CH_4_ modeling exercises, with or without using *δ* ^13^C‐CH_4_ as an additional constraint, have mostly focused on estimated emissions, we also recommend comparing different sink setups in models due to the large uncertainties associated with them.

### 4.2 Potential Explanations for the Failure of Decreasing [OH] to Track the Recent *δ* ^13^C‐CH_4_ Trend

We designed scenario J_[OH]− to test the hypothesis that the renewed growth of atmospheric CH_4_ after 2006 is due to a negative trend in atmospheric [OH] (Rigby et al., 2017; Turner et al., 2017). Since both studies inferred [OH] using MCF simulation with box models, a uniform [OH] for each of the N boxes was reported at each time step, where *N* is the number of boxes in their models. It was not possible to take those *N* values and implement them in a 3D model, which required the specification of a 3D structure in the OH field. Using those *N* scalars blindly in a 3D model (e.g., a single uniform [OH] when trying to reproduce a 1‐box model) does not yield CH_4_ lifetimes and gradients that are realistic (Naus et al., 2019). Therefore, instead of using the values of [OH] reported by Rigby et al. (2017) and Turner et al. (2017), we tested a hypothesis motivated by their conclusion that recent changes in CH_4_ were primarily due to decreasing atmospheric [OH]. Specifically, we tested the limiting case that all changes in atmospheric CH_4_ post‐2006 were due to changes in [OH], while CH_4_ emissions were constant at the average level from 2002 to 2006. We derived the necessary changes in [OH] by first calculating the required changes in lifetime to match observed CH_4_ growth given constant emissions each year post‐2006, then calculating the required changes in [OH] to produce those changes in lifetime assuming the other sink terms (Cl, O(^1^D), and soil sink) did not change. This yielded an ∼8% decrease in OH between 2007 and 2016, comparable to the [OH] trends proposed by Rigby et al. (2017) and Turner et al. (2017). By construction, this scenario matched the global CH_4_ growth rate (Figure 4a). However, this scenario produces a positive *δ* ^13^C‐CH_4_ trend contrary to atmospheric observations (Figure 5), which is the case for all combinations of emission scenario J_[OH]− with the three sink fractionation scenarios described in Section 2.5 (Figures 6 and 7, J_[OH]−). Instead, a negative trend in [OH] increased *δ* ^13^C‐CH_4_, which can be understood as follows.

All CH_4_ sink fractionation enriches the atmosphere with ^13^CH_4_ because the sinks preferentially consume ^12^CH_4_. While OH is the largest sink of atmospheric CH_4_, accounting for ∼86% of the total sink in our model, it is also the sink that fractionates the least. Therefore, if the OH sink weakens and the other sinks do not (as in scenario J_[OH]−), the resulting sink‐weighted fractionation in the atmosphere becomes stronger, which increases atmospheric *δ* ^13^C‐CH_4_. A ∼8% decrease in [OH] changes the sink‐weighted *ε* from −7.85‰ to −8.13‰ (in Equation 4). Turner et al. (2017) did not observe this behavior in their box models because they did not have sinks with different fractionations. Instead, they derived a change in the CH_4_ lifetime and attributed it all to a reduction in [OH]. With scenario J_[OH]−, we show that any such reduction in [OH] would increase atmospheric *δ* ^13^C‐CH_4_ contrary to observations after 2006. Fujita et al. (2020) also found a disagreement between the modeled and observed *δ* ^13^C‐CH_4_ when using the large reduction in [OH] from Turner et al. (2017) in a 3D model (NIES‐TM).

### 4.3 Uncertainty in Wetland Emissions

**Figure 8:**
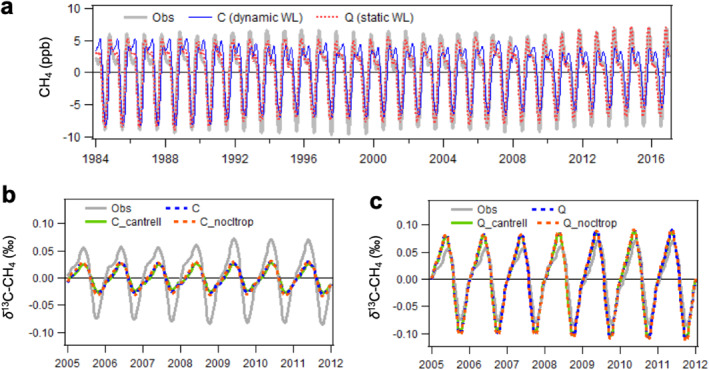
Modeled global Marine Boundary Layer mean CH_4_ (a) and *δ* ^13^C‐CH_4_ (b, c) seasonal cycles when using a dynamic WL map (scenario C) and a static WL map (scenario Q). In (b) and (c), “_cantrell” refers to the sink scenario using OH fractionation of −5.4‰ (Cantrell et al., 1990), while “_nocltrop” refers to the sink scenario excluding tropospheric Cl. Long‐term trends are first removed before estimating seasonal cycles by a 3‐year running average method.

The uncertainty of WL emissions is partially addressed in different emission scenarios with different annual WL emissions and different post‐2006 growth in latitudinal emissions, as discussed above (scenarios B_Mic+, C_WL+, D_trop_Mic+, E_trop_WL+, and M_more_trop_WL). To further evaluate the uncertainty of WL areas, we employ a substantially different WL area map in TEM and use those emissions in scenario Q_static_WL. These WL emissions are based on a static WL distribution (i.e., time invariant during the whole study period [Matthews & Fung, 1987]), and the seasonal and interannual variability of emissions is driven by meteorology in TEM. For WL emissions in all other emission scenarios, dynamic WL distributions are used, and the seasonal and interannual variability of emissions is driven by both seasonal and long‐term changes in WL inundation area (SWAMP‐GLWD data [Poulter et al., 2017]) and the meteorology in TEM. While annual WL emissions are similar between the two cases, the seasonal emission amplitude is 40% larger from emissions based on the static map than the dynamic map (Figure S6). WL emissions have the largest seasonal variability among all sources; this (Figure 8, Q_static_WL) produces larger seasonal cycles for atmospheric CH_4_ and *δ* ^13^C‐CH_4_ that can better match the observations. The simulated atmospheric CH_4_ seasonal cycles based on the dynamic map show a decreasing amplitude over time, which is inconsistent with observations. This is probably related to the long‐term decreasing trend in inundation area in the dynamic map.

Another significant difference between these two WL emissions is their latitudinal distributions, particularly in the Arctic (defined here as 60°N–90°N). We find that the Arctic WL emissions based on the dynamic map are less than 5 Tg/year, which are much lower than the cluster results from previous modeling studies (Saunois et al., 2016) and the 10 Tg/year from the static WL map. These yield considerable differences in modeled atmosphere CH_4_ and *δ* ^13^C‐CH_4_. Model‐observation agreement improves when using the static WL map (Figure 8), although this not always the case for locations further from the Arctic (not shown). Different latitudinal distributions in emissions also produce different emission‐weighted *δ* ^13^C‐CH_4_ signatures from WL due to spatial differences in WL *δ* ^13^C‐CH_4_ signatures. As a result, the global mean WL *δ* ^13^C‐CH_4_ signature is lower when using the WL emissions with more Arctic emissions where *δ* ^13^C‐CH_4_ signatures are more depleted. To account for this in the isotopic mass balance, we have increased ONG emissions by 20 Tg/year (i.e., 12% increase in total FE compared to those in the default sink scenario) and decreased ruminant emissions by the same amount in scenario Q_static_WL. From Figures 8b and 8c, we see that the differences in three sink scenarios have almost no impact on the simulated global mean *δ* ^13^C‐CH_4_ seasonal cycles, but the differences in WL maps play a dominant role. Simulated *δ* ^13^C‐CH_4_ seasonal cycles from SH Cape Grim site (Figure S5) also show only small differences when using three different sink setups.

The seasonal cycle of WL CH_4_ emissions based on the dynamic map is expected to be more reliable, due to a more realistic seasonal cycle in the inundation area. But the dynamic map provides lower Arctic emissions because the total area of WL in the Arctic is smaller in this map, which may be related to a limitation in the SWAMP data in identifying WL areas that are not inundated. Inundation areas are not necessarily equal to WL areas with CH_4_ emissions. Questions remain as to whether the significant long‐term decrease in inundation area, if it is true, represents a significant decrease in WL area and emissions. It is also possible that both maps miss WL areas or overestimate WL area fractions in some regions. Thus, a better quantification of WL area is essential to improve estimates of the CH_4_ budget. On the other hand, TEM has large uncertainties due to model parameters that cannot be fully evaluated with available data (Liu et al., 2020). Additional CH_4_ flux measurements, more accurate WL type, and area distribution information are required to improve the performance of process‐based WL models for future predictions (Liu et al., 2020).

### 4.4 Uncertainty in *δ* ^13^C‐CH_4_ Source Signatures

Sherwood et al. (2017) show a large spread in the probability distribution function (PDF) of global *δ* ^13^C‐CH_4_ signatures from most CH_4_ emission categories, which sometimes has been mistaken as a large uncertainty in their global mean source signature. In this study, we explore the spatial distribution of the *δ* ^13^C‐CH_4_ signatures and find that the spatial differences in *δ* ^13^C‐CH_4_ signatures are significant for FE, BB, and some Mic emissions that can partially explain the large spread in their source signature PDFs globally.

The spatial differences in *δ* ^13^C_FE_ signatures are mostly driven by the thermal maturity of the source rock and the presence of microbial activity where coal, oil, and gas are formed (Sherwood et al., 2017; Zumberge et al., 2012). For BB, ruminant, and wild animal emissions, the C_3_/C_4_ plant distribution dominates the spatial pattern of their *δ* ^13^C‐CH_4_ signatures (see discussion in SI Section 5). Field measurements of WL also indicate considerable latitudinal differences in WL *δ* ^13^C‐CH_4_ signatures with notably depleted signatures over the Arctic (Douglas et al., 2016; Fisher et al., 2017; Nakagawa et al., 2002), which may be caused by spatially different *δ* ^13^C‐CH_4_ signatures from soil organic carbon and different fractionation during methanogenesis, oxidation, and transport processes. Given that the CH_4_ sources and sinks are grossly underconstrained by observations, these broad spatial features in *δ* ^13^C‐CH_4_ are helpful in constraining the CH_4_ budget. However, arbitrarily selecting a value in the large spread of available *δ* ^13^C‐CH_4_ source signatures to represent global or regional means is not logical and can yield unrealistic interpretations on CH_4_ budget.

Here, we estimate the overall uncertainty in *δ* ^13^C‐CH_4_ source signatures in partitioning CH_4_ emissions by assigning grid‐level uncertainty to spatially resolved source signatures. For each source, we conduct 10,000 Monte Carlo (MC) simulations. Each MC simulation generates a new gridded signature map by randomly selecting a signature value from a Gaussian distribution defined by the gridded signature and its uncertainty as *μ* and *σ* for each grid cell. A new global mean signature is then calculated by weighting the new signature map by the gridded emission magnitude. As a result, we can robustly estimate the uncertainty in the global mean signature (Figure S4) that is constrained by the spatial pattern of emission (see SI for more details). The estimated uncertainties for the global weighted mean signatures (*σ* of 10,000 MC means) of ONG, coal, and geological seeps are 0.4‰, 1.1‰, and 1.5‰, respectively. The estimated uncertainties for global weighted mean signatures of WL, ruminants and wild animals, and BB are 0.06‰, 0.07‰, 0.14‰, respectively, which are considerably smaller than the uncertainties for the global mean signatures of waste/landfill, rice, and termite, whose spatial patterns of *δ* ^13^C‐CH_4_ signatures are not well known (Figure S4). Note that the assigned grid‐level uncertainty in *δ* ^13^C‐CH_4_ source signatures, albeit considerably large (SI Section 5), may not be able to fully account for systematic bias which are not well known from current measurements.

We evaluate the influence of the uncertainties of *δ* ^13^C‐CH_4_ signatures on emission partitioning using the mass balance equations of CH_4_ and *δ* ^13^C‐CH_4_ (see SI for details), since the emission partitioning from this approach is sufficient to reproduce observed global means of CH_4_ and *δ* ^13^C‐CH_4_ using the 3D model. The global mean FE magnitude is estimated to be 167 Tg/year, while the uncertainties in *δ* ^13^C‐CH_4_ signatures alone are estimated to account for a total uncertainty of 9.8 Tg/year in FE. In this study, we adopt geological seep emissions and their *δ* ^13^C‐CH_4_ signatures from Etiope et al. (2019), with a global total emission of 37 Tg/year and emission‐weighted mean signature of −47.9‰. However, a recent top‐down study based on ^14^CH_4_ measurements suggests a much smaller magnitude of natural geological CH_4_ emissions (∼1.6 Tg/year, Hmiel et al., 2020). Since the magnitude of natural fossil methane emissions is still debated (Etiope & Schwietzke, 2019), we assume an extreme case where the geological seep emission is zero to estimate this uncertainty. The resulting total FE, which is now composed of coal, ONG, and other energy emissions, is reduced by 15 Tg/year. This suggests that we would need to increase anthropogenic FE from 130 to 152 Tg/year if there is no contribution from geological seeps. Although this upward change in anthropogenic FE still seems reasonable compared with the different estimated FE magnitudes discussed above, this analysis does not evaluate the validity of low natural geo‐CH_4_ emissions but rather quantifies the change in anthropogenic FE to match the isotopic mass balance in a scenario in which geologic seep emissions are zero. It remains challenging to separate the natural FEs from anthropogenic FE based on global *δ* ^13^C‐CH_4_ mass balance, because the global mean signature of natural geo‐CH_4_ emissions (−47.9‰) is very close to those from anthropogenic FE (−45‰ to −43‰ range, see Figure S4c).

We should also recognize current limitations in understanding potential temporal changes in *δ* ^13^C‐CH_4_ source signatures due to limited availability of temporal information, except for the ONG signature in the U.S. Some temporal changes are expected due to changes in economic activity, which are mostly accounted for when changes in emissions are documented by the emission inventory. For example, the increased coal CH_4_ emissions in China (EDGAR 4.3.2, Janssens‐Maenhourt et al., 2019) enrich the global FF signatures; this is accounted for by multiplying the country‐level time‐invariant coal signatures, which are generally heavier in China than many other regions (Figure 3), with inventory coal CH_4_ emissions. For Mic sources such as WL and rice, their *δ* ^13^C‐CH_4_ signatures are subject to change since soil organic carbon, methanogenesis, soil/plant transport, and oxidation processes can be influenced by temperature, moisture content, etc. (Brownlow et al., 2017; Chanton, 2005; Fisher et al., 2017; Nakagawa et al., 2002). However, it is unclear whether these changes are apparent at decadal time scale. Current measurements of WL signatures are insufficient to identify a trend over the past decade. Thus, a pressing task is to increase measurements of *δ* ^13^C‐CH_4_ signature from natural sources and determine their spatiotemporal patterns. Temporal changes are also expected for global ruminant signatures due to changes in C_3_/C_4_ diet and the decreasing trend in atmospheric *δ* ^13^CO_2_ (a feature that can embed in plants); however, a recent analysis shows only marginal temporal change in ruminant *δ* ^13^C‐CH_4_ signatures due to a combination of these effects (Chang et al., 2019).

## 5 Conclusions and Future Work

Our study addresses different hypotheses that attempt to explain the post‐2006 increase in global atmospheric CH_4_ using a bottom‐up budgeting approach, atmospheric box‐modeling, and a limited set of atmospheric measurements. We first construct candidate emission and sink scenarios that can match atmospheric CH_4_ growth. A majority of the emissions scenarios are further modified to use the FE and Mic partitioning informed by global *δ* ^13^C‐CH_4_ mass balance. The FE and Mic partitioning is later confirmed by 3D tracer transport model (TM5) with reasonable agreements with the observed CH_4_ latitude gradients and global mean *δ* ^13^C‐CH_4_. Comparison of modeling performances from scenarios with different post‐2006 emissions provides further insights into the robustness of different hypotheses in explaining recent decreasing trends in global mean *δ* ^13^C‐CH_4_. This is the first study to comparatively test multiple hypotheses in one consistent model framework with full 3D modeling. We find that FF emissions based on the EDGAR 4.3.2 inventory, which show positive trends over time, do not agree with the observed *δ* ^13^C‐CH_4_ magnitude and long‐term trend because the mass balances of both CH_4_ and *δ* ^13^C‐CH_4_ are not satisfied. When a moderate positive trend is enforced for FF emissions, the mass balances require even more significant contributions from other processes that can reduce atmospheric *δ* ^13^C‐CH_4_, for example, decreasing BB emissions and/or decreasing soil sinks, together with a large increase in Mic emissions. This further discourages the proposition that FF emission increases are the dominant driver for the global CH_4_ increases after 2006 despite the possibility for small FF emission increases. We also find that a negative [OH] trend after 2006 with no change in emissions cannot track the observed decrease in global mean *δ* ^13^C‐CH_4_, contrary to previous studies (Rigby et al., 2017; Turner et al., 2017).

This study updates the large data set of *δ* ^13^C‐CH_4_ source signatures. Our model scenario using a global mean source signature for each emission category (Scenario H_mean_sig) yields modeled 3D fields that agree with the long‐term trend in global mean *δ* ^13^C‐CH_4_, confirming our emission partitioning based on the global mean source signatures and global mass balance approach. Our emission partitioning includes 167 Tg/year FE, 360–420 Tg/year Mic emissions (from 2000 to 2016), and ∼30 Tg/year BB (including biofuels) emissions over the study period in the default sink scenario. However, the updated source signature data set does show some regional differences compared with v2017 (Sherwood et al., 2017). Spatially resolved *δ* ^13^C‐CH_4_ source signature maps are developed in this study for ONG, coal, BB, and ruminant emissions, based on v2020 signature data set. They are used in a majority of model runs with additional spatially resolved signatures maps from WL (Ganesan et al., 2018) and natural geological seeps (Etiope et al., 2019). When comparing their performances with that from model scenario H_mean_sig, we find that spatial information of *δ* ^13^C‐CH_4_ source signature is important to match the observed latitudinal gradients and to further distribute emissions to different regions.

Large uncertainties remain in CH_4_ emissions and sinks, as demonstrated by the sensitivity analyses of the tropospheric Cl sink, OH sink fractionation, and WL areas and emissions. Through model comparisons with the observed global means and large‐scale latitudinal gradients of CH_4_ and *δ* ^13^C‐CH_4_, we can only confidently rule out a few hypotheses but cannot propose a best emission scenario. A few emission scenarios explaining the post‐2006 renewed growth of atmospheric CH_4_ seem equally plausible although they cannot match the observations perfectly. They include (i) increased emissions from microbial sources in the tropics (Nisbet et al., 2016, 2019; Schaefer et al., 2016); (ii) moderate increases in FF emissions and decreases in biomass burning emissions (Worden et al., 2017), though a smaller FF trend than proposed is required to better match *δ* ^13^C‐CH_4_ trend; and (iii) a significant decrease in soil sink (Ni & Groffman, 2018) accompanied by increases in WL emissions. Some inversion studies (Saunois et al., 2016) shift a considerable amount of NH emissions to the SH, especially to southern tropical WL emissions, to match the observed CH_4_ latitude gradient. But we find this adjustment has worsened the agreement with observed *δ* ^13^C‐CH_4_ gradients. Since the plausible emission scenarios still cannot perfectly match the observed CH_4_ latitude gradient, we need to shift emissions from the NH midlatitudes to the tropics or SH and increase N‐S *δ* ^13^C‐CH_4_ gradients at the same time. Thus, inversion study that can assimilate both CH_4_ and *δ* ^13^C‐CH_4_ is recommended to match both observed CH_4_ and *δ* ^13^C‐CH_4_ latitudinal gradients. These plausible emission scenarios can then serve as reasonable a priori in inverse modeling that includes *δ* ^13^C‐CH_4_ as a constraint to reduce spin‐up time.

While many CH_4_ modeling and global budget studies have mostly focused on estimating emissions, we also evaluate 3 different sink scenarios accompanied by 11 emission scenarios in the same model framework. We found that when using our default sink scenario including tropospheric Cl (∼13 Tg/year CH_4_ sink [Hossaini et al., 2016]) and OH fractionation of 3.9‰ (Saueressig et al., 2001), 167 Tg/year FE (assuming no temporal trend) can best match the atmospheric CH_4_ and *δ* ^13^C‐CH_4_ constraint combined with *δ* ^13^C‐CH_4_ source signatures. If we exclude the tropospheric Cl sink and increase OH sink to maintain similar total CH_4_ loss as the default sink scenario, we have to increase FE to 200 Tg/year (while ruminant emissions decrease by the same amount) to fit in the isotopic mass balance. If we use a OH fractionation factor of −5.4‰ (Cantrell et al., 1990) but still include tropospheric Cl, we find 135 Tg/year FE can best fit the new isotopic mass balance. Thus, we recommend evaluating sink setups in models due to the large uncertainties associated with them and additional research efforts to reduce these uncertainties.

In this study, we demonstrate that the long‐term globally distributed measurements of atmospheric *δ* ^13^C‐CH_4_ and *δ* ^13^C‐CH_4_ source signatures can help refine the CH_4_ budget, especially on the magnitude and spatial distribution of emissions. However, we should also acknowledge the uncertainties in *δ* ^13^C‐CH_4_ source signatures, which alone can account for a total uncertainty of 9.8 Tg/year in estimated FEs. To make full use of available *δ* ^13^C‐CH_4_ information for the studied regions, we should consider the spatial differences in *δ* ^13^C‐CH_4_ signatures and use regionally representative atmospheric measurements and sources signatures.

## Conflict of Interest

The authors declare no conflicts of interest relevant to this study.

## Supporting information

Supporting Information S1

## Data Availability

Atmospheric CH_4_ mole fraction data are provided by NOAA/OAR/Global Monitoring Laboratory, publically available at https://www.esrl.noaa.gov/gmd/ccgg/trends_ch4/. Atmospheric *δ* ^13^C‐CH_4_ data are provided by Institute of Arctic and Alpine Research (INSTAAR) of University of Colorado Boulder and updated for this study (Michel et al., 2021). They are publically available at https://doi.org/10.15138/79jq-qc24. A database of *δ* ^13^C‐CH_4_ source signature, v2020 (Sherwood et al., 2021), is compiled based on previously published data, and gridded maps are created from them as a result of this study. They are available at https://doi.org/10.15138/qn55-e011.
