# Supplementary material for: Improved Constraints on Global Methane Emissions and Sinks Using δ 13C‐CH4
Source: Global Biogeochem Cycles. 2021 Jun 17;35(6):e2021GB007000. doi: 10.1029/2021GB007000 (PMC8244052; doi:10.1029/2021GB007000)
Supplement: Supplementary file 1 — Supporting Information S1 [file GBC-35-e2021GB007000-s001.docx]

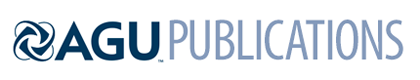


*Global Biogeochemical Cycles*

Supporting Information for

**Improved constraints on global methane emissions and sinks**

**using *δ*^13^C-CH_4_**

Xin Lan^1,2^, Sourish Basu^3,4^, Stefan Schwietzke^1,5^, Lori M. P. Bruhwiler^2^, Edward J. Dlugokencky^2^, Sylvia Englund Michel^6^, Owen A. Sherwood^6,7^, Pieter P. Tans^2^, Kirk Thoning^2^, Giuseppe Etiope^8,9^, Qianlai Zhuang^10^, Licheng Liu^10^, Youmi Oh^2,10^, John Miller^2^, Gabrielle Pétron^1,2^, Bruce H. Vaughn^6^, Monica Crippa^11^

^1^Cooperative Institute for Research in Environmental Sciences, University of Colorado Boulder, Boulder, CO, USA

^2^Global Monitoring Laboratory, National Oceanic and Atmospheric Administration, Boulder, CO, USA

^3^Earth System Science Interdisciplinary Center, University of Maryland, College Park, MD, USA

^4^Global Modeling and Assimilation Office, National Aeronautics and Space Administration

Goddard Space Flight Center, Greenbelt, MD, USA

^5^Environmental Defense Fund, Berlin, Germany

^6^Institute of Arctic and Alpine Research, University of Colorado Boulder, Boulder, CO, USA

^7^Department of Earth and Environmental Sciences, Dalhousie University, Canada

^8^Istituto Nazionale di Geofisica e Vulcanologia, Italy

^9^ Environmental Science and Engineering Department, Babes Bolyai University, Cluj-Napoca, Romania.

^10^Department of Earth, Atmospheric, and Planetary Sciences, Purdue University, West Lafayette, IN, USA

^11^Joint Research Centre, European Commission, Ispra, Italy

**Contents of this file**

Text section 1 to 5

Figures S1 to S6

Tables S1

**Introduction**

This document includes supporting figures and table referenced in the main manuscript and the supporting text. The supporting text includes 5 sections:

1. Details on isotopic mass balance equations
2. Atmospheric CH_4_ and *δ*^13^C-CH_4_ data and their uncertainties
3. Model details
4. Emission scenarios
5. Uncertainty in *δ*^13^C-CH_4_ source signature and its influence on emission partitioning

| Source | Total emissions | Spatial distribution | Seasonal cycle |
| --- | --- | --- | --- |
| Fossil Emission (FE) | EDGAR 4.3.2 for coal, oil and natural gas, and other energy/industry (Janssens-Maenhout et al., 2019; https://edgar.jrc.ec.europa.eu/overview.php?v=432_GHG ). ONG and coal are disaggregated because their *δ*^13^C-CH_4_ signatures are different. | | n/a (annual resolution) |
|  | Etiope et al. (2019) for geological seeps. | | Time invariant |
| Biomass and biofuel burning (BB) | Biomass burning fluxes between 1997-2016 are from GFED 4.1s with monthly resolution (Van der Werf et al., 2017) | | |
|  | Biomass burning fluxes before 1997 are from Reanalysis of the Tropospheric chemical composition project (Schultz et al., 2008) | GFED 4.1s for 2000 | GFED 4.1s for 2000 |
|  | Biofuel fluxes are from EDGAR 4.3.2 | | n/a (annual resolution) |
| Modern Microbial (Mic) | Ruminants and waste/landfills fluxes are from EDGAR 4.3.2 | | n/a (annual resolution) |
|  | Rice fluxes are from EDGAR 4.3.2 | | Matthews et al. (1991) with monthly resolution |
|  | Wild animal and termite fluxes are from Bergamaschi et al. (2007), with daily resolution but without inter-annual variability | | |
|  | Wetland (positive) and soil sink (negative) fluxes are from a process-based model with monthly resolution (Liu et al., 2019) | | |

**Table S1**. Data sources for emissions and their spatiotemporal patterns used in building different emission scenarios for model simulations


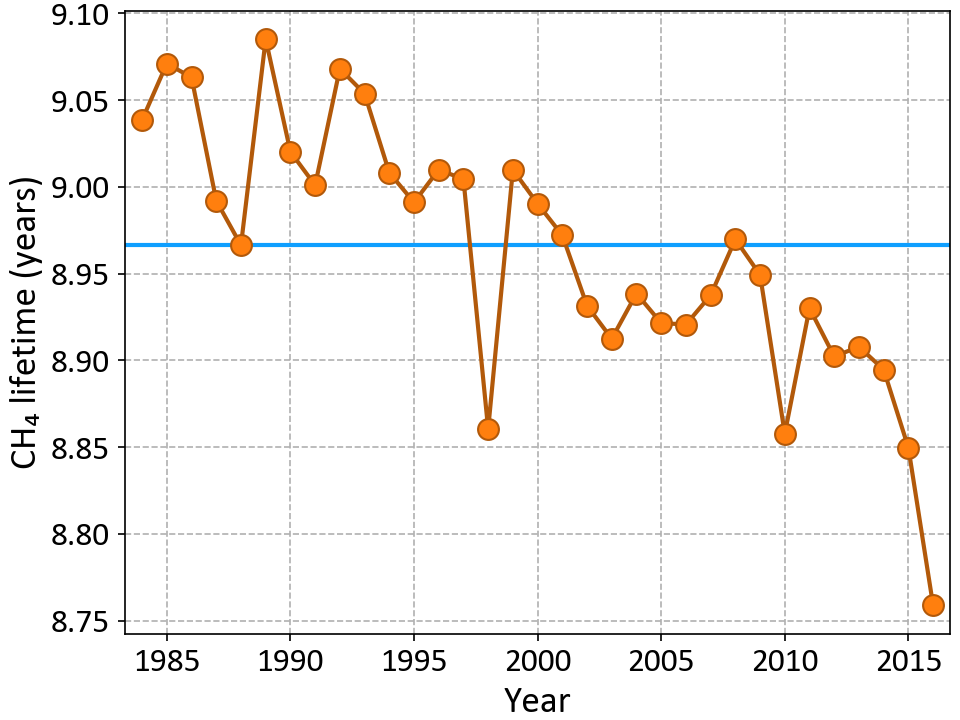


**Figure S1.** The lifetime of atmospheric CH_4_ from all loss processes in our base model setup. The orange circles are lifetimes consistent with CH_4_ loss in each year, whereas the blue line is the mean lifetime across 33 years.

**Figure S2** **a** Bottom-up CH_4_ emissions for each source category. Soil sink is combined with wetland emissions since it is negative (change from -29 to -37 Tg/yr from 1984 to 2016). **b** total emissions from bottom-up approach (i.e. the sum of those in **a**) and top-down approach using Eq. 1 (see section 2.4 for details).


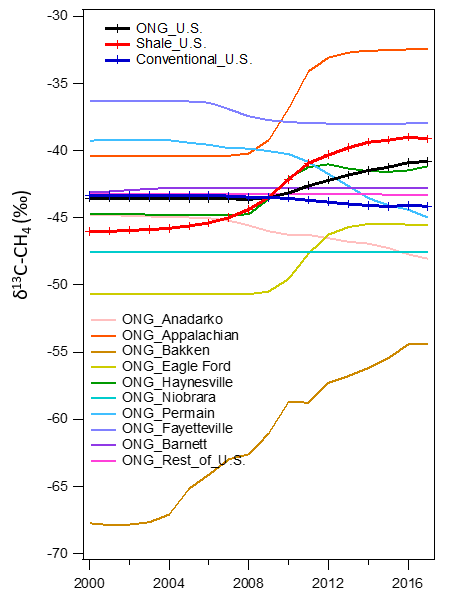


**Figure S3**. FF *δ*^13^C-CH_4_ signatures from the U.S. and its major ONG producing basins. The ONG signature is calculated as a weighted average (by production) of shale and conventional natural gas signatures. Based on updated FF signature from this study and the U.S. EIA statistics for production volumes by producing basins.
(https://www.eia.gov/naturalgas/weekly/).

**Figure S4**. Global mean *δ*^13^C-CH_4_ source signature and its uncertainty (upper panel of each subfigure), and time series of CH_4_ emission (bottom panel of each subfigure). CH_4_ emissions are from scenario C_WL+. Time series of global mean source signatures in **a**, **b**, and **c** are weighted by gridded emissions also from scenario C_WL+.


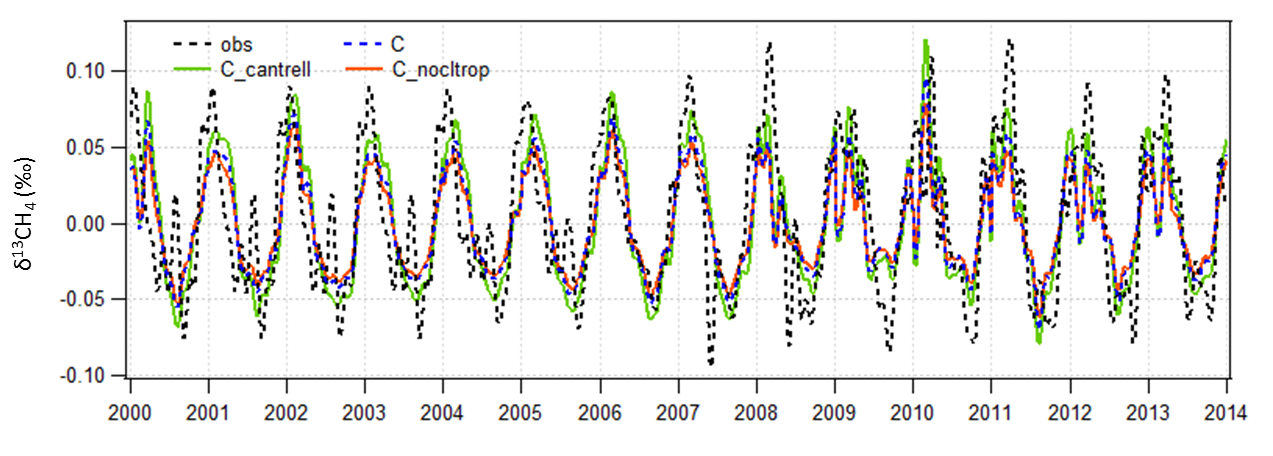


**Figure S5.** Observed and simulated seasonal cycles of *δ*^13^C-CH_4_ at Cape Grim Observatory (CGO), Australia (40.683⁰S, 144.690⁰E) in emission scenario C_WL+ combined with three different sink scenarios. ‘C_cantrell’ refers to the sink scenario using OH fractionation of -5.4‰, while ‘C_nocltrop’ refers to the sink scenario excluding tropospheric Cl. Long-term trends are first removed before estimating the seasonal cycles by a 3-year running average method.


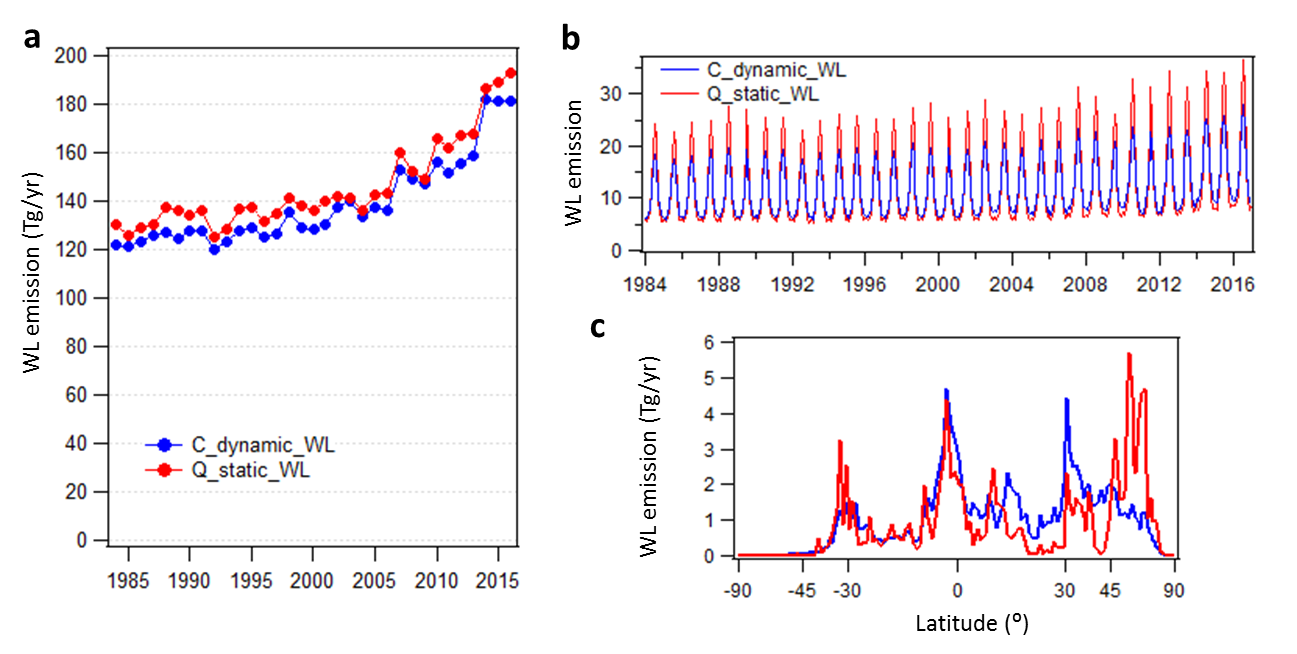


**Figure S6**. Comparisons of wetland (WL) emissions between static (red) and dynamic WL (blue) area maps for annual emissions (**a**), seasonal cycle (**b**) and latitude distribution (**c**).

**1. Details on isotopic mass balance equations**

We present here a more detailed description than section 2.3 for the mass balance equations of CH_4_ and *δ*^13^C-CH_4_, following Lassey et al. (2000) and Schwietzke et al. (2016), with minor revisions. These equations are the basis of our derived CH_4_ source partitioning when we design the modelled scenarios for TM5 runs, while the CH_4_ sinks are assumed known from TM5 sink set-ups (described in section 2.5). Later in this study, we confirm that TM5 modeled scenarios using these source partitioning yield good agreements with observed global mean CH_4_ and *δ*^13^C-CH_4._

Considering the global atmosphere as one box with mass conservation, the mass balance of CH_4_ can be expressed on a yearly time scale (t=1 yr) as

$$\frac{d\left[ \mathrm{CH}_{4} \right]}{\mathrm{dt}}=Q_{\mathrm{Atm}}-\frac{\left[ \mathrm{CH}_{4} \right]}{\tau} \text{SI-Eq.1}$$

where [CH_4_] is the global burden and τ is the atmospheric lifetime. Eq.1 indicates that the global atmospheric CH_4_ growth$\frac{d[\mathrm{CH}_{4}]}{\mathrm{dt}}$ is caused by the imbalance between total emissions to the atmosphere, $Q_{\mathrm{Atm}}$, and total sinks expressed as $\frac{\left[ \mathrm{CH}_{4} \right]}{\tau}$. In this study, τ was calculated using the sink fields in the TM5 model. While [CH4] is estimated by multiplying measured atmospheric CH_4_ (in ppb) with a conversion factor of 2.763 Tg/ppb that is based on TM5 atmospheric mass and sink distribution, we can calculate global total emissions $Q_{\mathrm{Atm}}$.

Similarly, the mass balance of *δ*^13^C-CH_4_ can be expressed as

$$\frac{d\left( \left[ \mathrm{CH}_{4} \right]R_{\mathrm{Atm}} \right)}{\mathrm{dt}}=Q_{\mathrm{Atm}}R_{Q}-\frac{\left[ \mathrm{CH}_{4} \right]}{\tau}R_{\mathrm{Atm}}\alpha\text{SI-Eq.2}$$

where $R_{x}$ is the isotopic ratio of ^13^C/^12^C of the emission source (Q) or the atmospheric reservoir (Atm). Different from Eq.1, there is a multiplying factor in the sink term (second term of the RHS). That is because of the sink fractionation effect. All CH_4_ sinks (OH, Cl, O(^1^D) and soil sink) enrich the atmosphere with ^13^C due to their faster reaction rate with ^12^C than with ^13^C. Thus, α is slightly smaller than 1 to account for the impact of the sink-weighted fractionation ($\varepsilon$) due to reactions with all sinks.

Following the product rule, we can write the right-hand side as:

$$\frac{d\left( \left[ \mathrm{CH}_{4} \right]{\cdot R}_{\mathrm{Atm}} \right)}{\mathrm{dt}}=\frac{d\left[ \mathrm{CH}_{4} \right]}{\mathrm{dt}}R_{\mathrm{Atm}}+\frac{dR_{\mathrm{Atm}}}{\mathrm{dt}}\left[ \mathrm{CH}_{4} \right] \text{SI-Eq.3}$$

Combining SI-Eq.2 and SI-Eq.3 yields:

$$Q_{\mathrm{Atm}}R_{Q}-\frac{\left[ \mathrm{CH}_{4} \right]}{\tau}R_{\mathrm{Atm}}\alpha=\frac{d\left[ \mathrm{CH}_{4} \right]}{\mathrm{dt}}R_{\mathrm{Atm}}+\frac{dR_{\mathrm{Atm}}}{\mathrm{dt}}\left[ \mathrm{CH}_{4} \right]$$

And with ${R_{Q}}/{R_{\mathrm{reference}}}={\delta^{13}C}_{Q}+1$ and ${R_{\mathrm{Atm}}}/{R_{\mathrm{reference}}}={\delta^{13}C}_{\mathrm{Atm}}+1$ where ${\delta^{13}C}_{Q}$ is the combined signal of *δ*^13^C from all sources emitted to the atmosphere:

$$Q_{\mathrm{Atm}}\left( {\delta^{13}C}_{Q}+1 \right)R_{\mathrm{reference}}-\frac{\left[ \mathrm{CH}_{4} \right]}{\tau}\left( {\delta^{13}C}_{\mathrm{Atm}}+1 \right)R_{\mathrm{reference}}\alpha=\frac{d\left[ \mathrm{CH}_{4} \right]}{\mathrm{dt}}\left( {\delta^{13}C}_{\mathrm{Atm}}+1 \right)R_{\mathrm{reference}}+\frac{d\left( \left( {\delta^{13}C}_{\mathrm{Atm}}+1 \right)R_{\mathrm{reference}} \right)}{\mathrm{dt}}\left[ \mathrm{CH}_{4} \right]$$

Since $R_{\mathrm{reference}}$ is a constant, the equation above simplifies to:

$$Q_{\mathrm{Atm}}\left( {\delta^{13}C}_{Q}+1 \right)-\frac{\left[ \mathrm{CH}_{4} \right]}{\tau}\left( {\delta^{13}C}_{\mathrm{Atm}}+1 \right)\alpha=\frac{d\left[ \mathrm{CH}_{4} \right]}{\mathrm{dt}}\left( {\delta^{13}C}_{\mathrm{Atm}}+1 \right)+\frac{d{\delta^{13}C}_{\mathrm{Atm}}}{\mathrm{dt}}\left[ \mathrm{CH}_{4} \right]$$

From where:

$${\frac{d{\delta^{13}C}_{\mathrm{Atm}}}{\mathrm{dt}}\left[ \mathrm{CH}_{4} \right]=Q}_{\mathrm{Atm}}\left( {\delta^{13}C}_{Q}+1 \right)-\left( {\delta^{13}C}_{\mathrm{Atm}}+1 \right)\left( \frac{\left[ \mathrm{CH}_{4} \right]}{\tau}\alpha+\frac{d\left[ \mathrm{CH}_{4} \right]}{\mathrm{dt}} \right)\text{ }\text{SI-Eq.4}$$

Using SI-Eq.1 to substitute $\frac{\left[ \mathrm{CH}_{4} \right]}{\tau}$ in SI-Eq.4 gives:

$${\frac{d{\delta^{13}C}_{\mathrm{Atm}}}{\mathrm{dt}}\left[ \mathrm{CH}_{4} \right]=Q}_{\mathrm{Atm}}\left( {\delta^{13}C}_{Q}+1 \right)-\left( {\delta^{13}C}_{\mathrm{Atm}}+1 \right)\left( \left( Q_{\mathrm{Atm}}-\frac{d\left[ \mathrm{CH}_{4} \right]}{\mathrm{dt}} \right)\alpha+\frac{d\left[ \mathrm{CH}_{4} \right]}{\mathrm{dt}} \right)$$

Using $\alpha=1+\varepsilon$ and dividing both the left-hand and right-hand sides by $Q_{\mathrm{Atm}}$ yields:

$$\frac{d{\delta^{13}C}_{\mathrm{Atm}}}{\mathrm{dt}}\frac{\left[ \mathrm{CH}_{4} \right]}{Q_{\mathrm{Atm}}}=\left( {\delta^{13}C}_{Q}+1 \right)-\frac{\left( {\delta^{13}C}_{\mathrm{Atm}}+1 \right)}{Q_{\mathrm{Atm}}}\left( \left( Q_{\mathrm{Atm}}-\frac{d\left[ \mathrm{CH}_{4} \right]}{\mathrm{dt}} \right)\left( 1+\varepsilon\right)+\frac{d\left[ \mathrm{CH}_{4} \right]}{\mathrm{dt}} \right)=\left( {\delta^{13}C}_{Q}+1 \right)-\frac{\left( {\delta^{13}C}_{\mathrm{Atm}}+1 \right)}{Q_{\mathrm{Atm}}}\left( Q_{\mathrm{Atm}}\left( 1+\varepsilon\right)-\varepsilon\frac{d\left[ \mathrm{CH}_{4} \right]}{\mathrm{dt}} \right)=\left( {\delta^{13}C}_{Q}+1 \right)-\left( {\delta^{13}C}_{\mathrm{Atm}}+1 \right)\left( 1+\varepsilon\right)+\frac{\left( {\delta^{13}C}_{\mathrm{Atm}}+1 \right)}{Q_{\mathrm{Atm}}}\varepsilon\frac{d\left[ \mathrm{CH}_{4} \right]}{\mathrm{dt}}={\delta^{13}C}_{Q}-{\delta^{13}C}_{\mathrm{Atm}}\left( 1+\varepsilon\right)-\varepsilon+\varepsilon\frac{\left( {\delta^{13}C}_{\mathrm{Atm}}+1 \right)}{Q_{\mathrm{Atm}}}\frac{d\left[ \mathrm{CH}_{4} \right]}{\mathrm{dt}}$$

Using $\alpha=1+\varepsilon$ on the second term of the right-hand side gives:

$$\frac{d{\delta^{13}C}_{\mathrm{Atm}}}{\mathrm{dt}}\frac{\left[ \mathrm{CH}_{4} \right]}{Q_{\mathrm{Atm}}}={\delta^{13}C}_{Q}-{\delta^{13}C}_{\mathrm{Atm}}\alpha-\varepsilon+\varepsilon\frac{\left( {\delta^{13}C}_{\mathrm{Atm}}+1 \right)}{Q_{\mathrm{Atm}}}\frac{d\left[ \mathrm{CH}_{4} \right]}{\mathrm{dt}}$$

Rearranging the above equation yields:

$${\delta^{13}C}_{Q}= \alpha{\delta^{13}C}_{\mathrm{Atm}}+\varepsilon-\varepsilon\frac{\left( {\delta^{13}C}_{\mathrm{Atm}}+1 \right)}{Q_{\mathrm{Atm}}}\frac{d\left[ \mathrm{CH}_{4} \right]}{\mathrm{dt}}+\frac{d{\delta^{13}C}_{\mathrm{Atm}}}{\mathrm{dt}}\frac{\left[ \mathrm{CH}_{4} \right]}{Q_{\mathrm{Atm}}} \text{SI-Eq.5}$$

SI-Eq.5 describes the exact formulation between ${\delta^{13}C}_{Q}$ and ${\delta^{13}C}_{\mathrm{Atm}}.$ It is arranged in this form because if the atmosphere is in ^13^C/^12^C and CH_4_ steady state, i.e., $\frac{d[\mathrm{CH}_{4}]}{\mathrm{dt}}=0$ and $\frac{d{\delta^{13}C}_{\mathrm{Atm}}}{\mathrm{dt}}=0,$ it is obvious that SI-Eq.5 becomes

$${{{\delta^{13}C}_{Q}=\alpha\cdot\delta}^{13}C}_{\mathrm{Atm}}+\varepsilon\text{SI-Eq.6}$$

In our study, the total sink-weighted average fractionation factor is calculated as:

$$\varepsilon=\frac{\sum S_{i}\varepsilon_{i}}{\sum S_{i}}$$

where $S_{i}$ is the individual CH_4_ sink strength and $\varepsilon_{i}$ is its fractionation factor defined as (k_13_/k_12_ – 1) for CH_4_ reactions with the sink *i* (k_13_ is the reaction rate constant for ^13^C while k_12_ is the reaction rate constant for ^12^C). Individual CH_4_ sink strengths $S_{i}$ are assumed known from the sink set-up in TM5 that is described in section 2.5. ${\delta^{13}C}_{Q}$ is estimated using SI-Eq. 5 with known $Q_{\mathrm{Atm}}$ from SI-Eq.1. ${\delta^{13}C}_{\mathrm{Atm}}$ comes from atmospheric measurements. The steady state approximation in SI-Eq.6 (Eq. 4 in the main text) yields about 0.3‰ difference between the estimated${\delta^{13}C}_{Q}$ in SI-Eq.5 and SI-Eq.6.

Total emissions to the atmosphere $Q_{\mathrm{Atm}}$ include sub-categories of emissions (Q) from microbial (Mic) which are composed of emissions from wetland and Ag/waste emissions including rice, ruminants, wild animals, termites and waste/landfills sources, fossil emission (FE, including FF and natural geological seeps) and biomass/biofuel burning (BB) sources as

$Q_{\mathrm{Atm}}=Q_{\mathrm{Mic}}+Q_{\mathrm{FE}}+Q_{\mathrm{BB}} \text{SI-Eq.7}$

A similar equation can also be written for ${\delta^{13}C}_{\mathrm{atm}}$

${{\delta^{13}C}_{Q}\cdot Q}_{\mathrm{Atm}}={\delta^{13}C}_{\mathrm{Mic}}\cdot Q_{\mathrm{Mic}}+{\delta^{13}C}_{\mathrm{FE}}\cdot Q_{\mathrm{FE}}+{\delta^{13}C}_{\mathrm{BB}}\cdot Q_{\mathrm{BB}} \text{SI-Eq.8}$

where ${\delta^{13}C}_{x}$in the right-hand side is the emission-weighted source signature of a specific category of emissions. ${\delta^{13}C}_{x}$ are available from our source signature database. Thus, we can solve for $Q_{\mathrm{FE}}$and $Q_{\mathrm{Mic}}$ when we assume known $Q_{\mathrm{BB}}$.

**2. Atmospheric CH_4_ and *δ*^13^C-CH_4_ data and their uncertainties**

Uncertainties are reported for each CH_4_ measurement based on analytical repeatability, reproducibility, and our ability to propagate the WMO X2004A CH_4_ mole fraction standard scale. Analytical repeatability is based on approximately monthly measurements of target tanks, the relative stability of the standard or reference gas aliquots during each day of flask-air measurements, or on the average absolute value of agreement between pairs of samples collected nearly simultaneously. It varies with analytical instrument from 0.2 to 2.3 ppb, and improved repeatability reflects the improvement of analytical techniques over time. Propagation of the scale is based on the reproducibility determined for scale propagation in our calibration laboratory. It has a fixed value of 0.5 ppb based on subsequent calibrations of the same cylinder at least one year after the first. Reproducibility is based on long-term variations in measurements of target cylinders, typically ~0.3 ppb. The three terms are added in quadrature (square root of the sum of the squares) to estimate the measurement uncertainty at 68% confidence interval. Uncertainty of global mean CH_4_ are estimated using non-parametric statistical methods that vary the network distribution (through bootstrap approach, Steele et al., 1992), and include analytical uncertainty (Dlugokencky et al., 1994). Uncertainty of global mean CH_4_ generally falls in the range of 0.4 to 0.8 ppb.

The mass balance calculations and TM5 simulations start in 1984, though atmospheric *δ*^13^C-CH_4_ is only available from NOAA/INSTAAR starting at 1998. For 1984-1998, we use the global average atmospheric *δ*^13^C-CH_4_ data from Schaefer et al. (2016), which increase by ~ 0.4‰ during this period. These data were adjusted to the NOAA/INSTAAR *δ*^13^C-CH_4_ scale. For NOAA/INSTAAR *δ*^13^C-CH_4_ measurements, measurements of surveillance cylinders alongside with samples validate the stability of the NBS-19 scale (Tyler 1986). Daily surveillance cylinders allow for monitoring of accuracy and precision of daily *δ*^13^C-CH_4_ measurements. Analytical uncertainty for individual measurement is typically better than 0.065‰, which is calculated as the 2-week standard deviation of the daily surveillance cylinder at the time that the air sample is analyzed. This number is also consistent with the measurement repeatability estimated from pair differences of flasks from our marine boundary layer (MBL) sites (~ 0.05‰ for almost all MBL sites). The long-term data from surveillance cylinder is used to quantify long-term reproducibility in measurement. In this dataset, we used the long-term surveillance cylinder data to evaluate whether some data are outside the long-term target and flag the data in runs where the surveillance cylinder is 0.24 ‰ higher or lower than its long-term mean. Several daily sample runs were flagged because the samples have a disproportionate influence on the global mean *δ*^13^C-CH_4_.

Uncertainties of global mean *δ*^13^C-CH_4_ and their latitudinal gradients are estimated by accounting for network distribution, analytical and atmospheric uncertainties, and bias uncertainties. To calculate each uncertainty, we use bootstrap method to create 100 realizations of the MBL surface. MBL surface is calculated using data extension and integration (DEI) methods developed by Masarie and Tans (1995). The network uncertainty imagines alternative networks (while retaining sites in different latitude bins). Analytical and atmospheric uncertainties include uncertainties caused by synoptic atmospheric variability and the combined measurement and sampling handling uncertainties. It is quantified by bootstrapping the measurements from the MBL sites by randomly adding noise to the data but keeping the monthly average of the residuals the same. Residual is the difference between the data and the smooth curve which is composed of long-term trend, interannual variability and seasonal cycle (Thoning et al., 1989). We aim to mimics the synoptic variability encompassing measurement and sampling handling uncertainty. The results are in the range of 0.02‰ to 0.035‰ on approximately weekly global mean δ^13^C-CH_4_. The bias uncertainty accounts for unknown bias in measurement by randomly assigning error for variable amounts of time. This is necessary because the variance of trap data over longer time periods decreases more slowly than N^-1^, indicating the existing of a bias component. The bias range of 0.012‰ is taken from 3-month smoothed curves of the daily surveillance cylinder. The uncertainty of each component is the standard deviation of the 100 bootstraps at each time step and are added in quadrature to represent the total uncertainty. For the latitude gradients, the uncertainties are the standard deviation of each of the 41 binned latitudes for each of the 100 DEI surfaces for each component uncertainty.

**3. Modeling details**

In this study, we modeled the OH sink by scaling the OH field of Spivakovsky et al. (2000) by 0.901 when a tropospheric Cl sink of CH_4_ from Hossaini et al (2016) was applied. In another sink scenario without a tropospheric Cl sink, the OH field of Spivakovsky et al. (2000) was scaled by 0.9255 to ensure similar long-term CH_4_ loss across the two sink scenarios. Both scaling factors are within the bounds specified by Montzka et al (2011) to be consistent with the observed long-term decay of atmospheric MCF. For each of these chemical sinks of CH_4_, fractionation factors for each of the reactions were applied to separately simulate the destruction of ^13^CH_4._ In our default sink scenario, we used -3.9‰ and -13‰ for the fractionations in reactions with OH and O(^1^D), respectively (Saueressig et al., 2001). For reaction with Cl, we used the temperature-dependent form prescribed in Table 2 of Saueressig et al. (1995). We modeled the soil sink as a first-order reaction affecting only the surface layer of TM5:

$$\frac{d\left[ X_{\mathrm{CH}_{4}} \right]}{\mathrm{dt}}={-k}_{\mathrm{soil}}\left[ X_{\mathrm{CH}_{4}} \right]\cdot soil\_sink\_map$$

where soil_sink_map is the spatiotemporally varying soil sink flux map from TEM (Liu et al., 2020), ${[X}_{\mathrm{CH}_{4}}$] is the surface layer mole fraction of CH_4_, and k_soil_ is a constant calculated to ensure that the annual total loss due to this reaction is equal to the annual total sink from the soil sink map. Since the sink formulated this way depends on the CH_4_ mole fraction field in the atmosphere, the global total sink calculated this way is not exactly equal to the total soil sink from the TEM flux map for all scenarios. However, the difference between the total loss from this reaction and the total soil sink from TEM is under 0.5 Tg CH_4_/year for all years, much smaller than the uncertainty on the soil sink. This formulation of the soil sink as a reaction rather than a negative flux allows us to implement fractionation for the ^13^CH_4_ soil sink by modifying k_soil_ by the relevant fractionation factor [King et al. 1989]. We note here that k_soil_ derived this way depends on the transport model geometry, specifically our choice of running TM5 globally at 6°x4° with 25 vertical layers, and will need to be re-derived for a different transport model setup.

For each year, the CH_4_ lifetime was calculated as 1/log(C_0_/C_1_), where C_0_ (C_1_) was the total atmospheric burden of this tracer in the model at the beginning (end) of the year. Despite the climatological OH, Cl and O(^1^D) fields, the modeled CH_4_ lifetime in the model is not a constant every year from 1984 to 2016 due to changing covariances between interannually varying meteorology and a climatological OH distribution (Figure S1). As mentioned in section 2.4, the total CH_4_ emissions for each year were adjusted to be consistent with the observed CH_4_ global mean growth rate in the MBL (https://www.esrl.noaa.gov/gmd/ccgg/trends_ch4/) and the CH_4_ lifetime for that year (i.e. the Eq.1), using a global average conversion of 2.763 Tg CH_4_/ppb relevant to our TM5 model.

For model spin up, we try to use a realistic initial condition because the better the initial condition, the less time needed for spin up. we constructed the initial CH_4_ mole fraction field on Jan 1, 1984 as follows. First, we took the 3D CH_4_ mole fraction field from CarbonTracker-CH_4_ (CT-CH_4_) on Jan 1, 2003 (https://www.esrl.noaa.gov/gmd/ccgg/carbontracker-ch4/). Since the CT-CH_4_ assimilation started on Jan 1, 2000, its posterior mole fraction field after 3 years is expected to have spatial gradients consistent with CH_4_ observations globally. Next, we calculated the average Pacific Ocean MBL CH_4_ mole fraction from this field by considering the lowest ~160 hPa between 180°W and 174°W, and derived a scaling factor between this calculated quantity and the January 1984 observed MBL average CH_4_ from NOAA’s global network (https://www.esrl.noaa.gov/gmd/ccgg/trends_ch4/). We scaled the Jan 1, 2003 CT-CH_4_ field by this factor to arrive at a CH_4_ field for January 1984 that was consistent with observed MBL CH_4_ and its latitudinal gradient and had vertical gradients consistent with the TM5 model. We then calculated the initial ^13^CH_4_ field in January 1984 from this initial CH_4_ field and the estimated global average *δ*^13^C-CH_4_ of -47.501‰ in 1984 [Schaefer et al. (2016); Schwietzke et al (2016)]. The “time scales” of 1, 5, and 9 years discussed in main text are one e-folding time. Since the initial conditions in CH_4_ and ^13^CH_4_ are realistic, then 16 years of spin up time is be sufficient.

**4. Emission scenarios**

Section 2.4 includes key information on the emission scenario design. This SI section provides some additional details. Some emission inventories do not fully cover our study period of 1984-2016. Emission data from the EDGAR 4.3.2 inventory, which are only available until 2012, are extended to 2016 using 2012 emission maps for 2013-2016. For BB, GFED 4.1s inventory covers 1997-2016. Before 1997, we scale the GFED emission maps for 2000 to the total annual emissions from the Reanalysis of the Tropospheric chemical composition project (RETRO, Schultz et al., 2008), to avoid inconsistencies in spatial distributions between RETRO and GFED maps.

As discussed in section 3.1, the *δ*^13^C-CH_4_ mass balance requires a significant upward adjustment in total fossil emission magnitudes compared to the EDGAR 4.3.2 inventory emissions. For most scenarios (except A_FF+), we assign all required changes in FF emissions to ONG emissions, leaving the coal emissions unchanged. That is because the required adjustment is relatively large (40 Tg/yr to 15 Tg/yr from 1984 to 2016) compared with the absolute magnitude of coal emissions (20 to 40 Tg/yr from 1984 to 2016). So, it seems less likely that coal emissions should be increased to fill the gap. It is also evident that emission inventories tend to underestimate the magnitude of ONG emissions based on measurements from major oil and gas production regions (for example: Brandt et al., 2014; Alvarez et al., 2018). Given the coal and ONG emissions have relatively similar δ^13^C-CH_4_ signature (within 1‰ difference, as shown in Fig. S4), the impacts of different attributions of increased emissions to coal versus ONG on the global *δ*^13^C-CH_4_ trend, if included in our study, would still be difficult to separate.

For Scenario C_WL+, we assume wetland (WL) emission increases are fully responsible for the global emissions increase since 2007. Starting from 2007, changes in the global budget relative to 2006 are applied to the WL emissions. Thus the variability in global budget is propagated to the resulting WL emissions, which shows large increases in WL emissions in 2007 and 2014 (Fig. S6a). Ag/waste emissions are scaled slightly to fit the global budget.

For scenario D_trop_Mic+, we assign increases in WL, rice and ruminant emissions by latitudinal zones according to Nisbet et al. (2019). By assuming these 3 categories are responsible for post-2006 increases in CH_4_ emissions, we add the emission increases since 2007 relative to 2000-2005 mean to each category with the same proportional increase for all 3 categories. As a result, there are significant increases in tropical microbial emissions since 2007, especially from 0-30°S. The interannual increases from Nisbet et al. (2019) are used after being scaled down (slightly) by multiplying a factor determined from 2000-2005 mean emission from their model relative to the 2000-2005 mean from our model, because global total emissions from their study are ~ 70 Tg/yr greater than from our model, and their study assumes constant [OH] without interannual variability. Waste/landfill emissions are kept the same as in scenario C_WL+ and E_trop_WL+ (see below). After the added increases, we scale the ruminant emissions again to close the global budget. Scenario D_trop_Mic+ has a slightly larger increase in WL emissions compared with scenario B_Mic+.

For scenario E_trop_WL+, we assign increases only in WL emissions by latitudinal zone according to Nisbet et al. (2019). Increases in total emissions since 2007 relative to the 2000-2005 mean are added to our process-based WL emissions, assuming the increase in WL emissions is solely responsible for post-2006 global emission increases. As a result, there are significant increases in tropical WL emissions since 2007, especially from 0-30°S. Similarly, the interannual increases from Nisbet et al. (2019) are used after scaling them (slightly down) by multiplying a factor calculated from 2000-2005 mean emission from their model relative to the 2000-2005 mean from our model. Ag/waste emissions are scaled to fit the global budget. Scenario E_trop_WL+ is similar to C_WL+ in category emissions, except for the distribution of WL over latitude zones after 2007. Before 2007, emissions in E are exactly the same as those in C_WL+.

For scenario G_soil-, we assign a ~77% decline in soil sink over 1988-2015 (soil sink changed from 31 Tg/yr to 7 Tg/yr with inter-annual variability), according to Ni and Groffman (2018). Our annual soil sink matches Ni and Groffman (2018) Fig 2A for the annual mean values, which are a direct average of limited data from different locations around the global from each year. Our soil sink maps are scaled globally to match their global mean soil sink, i.e., we use the global total emissions from Ni and Groffman (2018) only, but the spatial patterns are still from our process-based model. We cannot scale the soil sink for 0-60°N only, although the decline was attributed mostly from this latitude zone in their study. That would have produced positive soil sink values for some grids. FE in this scenario is the same as in scenario C_WL+. WL emissions are from our bottom-up estimates. Ag/waste emissions are scaled to fit the global budget, which results in only small changes (vary by year, but generally < 10 Tg/yr) compared with original annual Ag/waste emissions from EDGAR 4.3.2. Total Ag/waste increase is adjusted to 19 Tg/yr from 1999-2006 mean to 2016 level, slightly lower than the 22 Tg/yr from EDGAR 4.3.2. The soil sink for 1984-1987 was not changed.

**5. Uncertainty in *δ***^13^**C-CH**_4_ **source signature and its influence on emission partitioning**

The uncertainty of global weighted mean WL signature is estimated using 10,000 Monte Carlo (MC) simulations with grid-level *δ*^13^C-CH_4_ signatures and emissions. Each MC simulation samples randomly from a constructed Gaussian distribution defined by the signature value and its uncertainty as μ and σ for each grid cell. The uncertainty of *δ*^13^C-CH_4_ signatures is estimated from available WL studies in the 2020v dataset documenting a total of 981 WL samples. The WL signature uncertainties are considerably large and dependent on latitude bin (90⁰S to 0⁰: 2.2‰; 0⁰ to 15⁰N: 2.6‰; 15⁰N to 55⁰N: 2.4‰; 55⁰N to 90⁰N: 4.5‰) to account for the variability of available *δ*^13^C-CH_4_ samples. The assumption of a Gaussian distribution allows the MC procedure to sample outside σ to enlarge the spread of the final uncertainty estimates. In each MC simulation, a new global mean signature is calculated by weighting the new gridded signature map by emission map. We use emission scenario C_WL+ as the base scenario to estimate the uncertainty in *δ*^13^C-CH_4_ source signatures. The resulting global mean WL signatures are different for each year due to annual variations in the emission map. However, the resulting uncertainty of the yearly global weighted mean WL signatures, as the σ of 10,000 MC weighted means, is generally small (~ 0.055‰, Figure S4).

The spatial distribution of *δ*^13^C-CH_4_ signatures for both ruminant and biomass burning are dependent of the *δ*^13^C-CH_4_ signatures in the source materials, and thus related to the C_3_/C_4_ plant distribution. The ^12^C/^13^C content of CH_4_ emitted from biomass burning has been measured to be consistent with the fuel burned (Snover et al., 2000). Following Schwietzke et al. (2016), the C_3_/C_4_ emission distributions (as the C_3_/C_4_ fraction of each grid cell) are estimated by the average of C_3_/C_4_ distribution map from Still et al. (2003) and GFED1 fire emission map (Randerson et al., 2012), which are assumed to be time-invariant in our study. For biomass burning the mean C_3_ and C_4_ plant signatures are estimated from 825 modern plants (Cerling et al., 1997), which are -12.5±1.1‰ and -26.7±2.3‰, respectively. For a given grid cell, the *δ*^13^C-CH_4_ signature is calculated as the weighted average of the C_3_ and C_4_ signatures with their relative fractions. The grid-level uncertainty is estimated as the total uncertainty induced by the uncertainty of the mean signatures, and the uncertainty of the C_3_/C_4_ plant distributions, which is estimated as the difference in *δ*^13^C-CH_4_ when using two different C_3_/C_4_ plant distribution maps. As a result, the grid-level uncertainty ranges from 1.1 to 14.3‰ across different grid cells. Then 10,000 MC simulations are conducted to estimate the uncertainty of the global weighted mean BB signatures, as done for WL. We assume the same *δ*^13^C-CH_4_ signatures for biofuel emissions as biomass burning emissions. For ruminants and wild animals, the *δ*^13^C-CH_4_ signatures are based on 227 samples in our v2020 database, which averaged to -67.8±2.1‰ and -54.5±1.7‰ for C_3_- and C_4_- fed animals, respectively. The grid-level mean and uncertainty, and the global weighted mean signatures are then estimated in the same way as for biomass burning emissions. The resulting grid-level uncertainty ranges from 1.5 to 13.4‰ across grid cells. See Figure S4 for the global weighted mean signatures and their uncertainties.

For waste/landfills, termite and rice emissions, the spatial distributions of their *δ*^13^C-CH_4_ signatures are not well defined from available measurements, thus their global mean signatures are used for each grid cell in our 3D model. The global mean and its uncertainty are directly estimated using the mean of the *δ*^13^C-CH_4_ signature in each study documented in the 2020v dataset and its uncertainty, which mostly reflects measurement uncertainty. We also use 10,000 MC simulations. For rice emissions, a total of 360 signatures from 18 geographic regions and studies are available from our database. For each MC simulation, we create a new set of 18 studies by bootstrapping the original 18 studies with random selection that allows repetition [*Diaconis and Efron*, 1983]. For each of the new 18 studies, a new mean signature is selected from the Gaussian distribution defined by the original study mean and its uncertainty. For each MC simulation, the global mean signature is the mean of the new means from the 18 new studies. After 10,000 MC simulations, we get a robust estimate of the global mean as the mean of 10,000 global means, and its uncertainty as the σ of 10,000 global means. For waste/landfills, 179 *δ*^13^C-CH_4_ signatures from 17 geographic regions and studies are available from our database. For termites, 29 *δ*^13^C-CH_4_ signatures from 6 geographic regions and studies are available. The same approach used for the rice emissions is used for estimating the global mean waste/landfills and termite signatures and their uncertainties. The resulting global means for rice, waste/landfills, and termites are -60.5±1.1‰, - 55.6 ±1.7‰, and 63.4±2.8‰, respectively. Their uncertainties are generally much larger compared with those source categories (Figure S4) whose spatial distributions of *δ*^13^C-CH_4_ signatures are better defined.

For fossil emissions, the gridded map of *δ*^13^C-CH_4_ signatures is created based on the spatial distribution of *δ*^13^C-CH_4_ signatures. Since for most cases the country-level mean signatures are used to represent all grid cells for that country, the grid-level uncertainty is assigned as the standard deviation of all available samples within the country. The uncertainty of the global weighted mean signatures is also estimated using 10,000 MC simulations by sampling from a constructed Gaussian distribution for each grid cell, as done for WL emissions. For coal emissions, the country means range from -30.8‰ to -64.1‰, while the grid-level uncertainty, i.e., the assumed σ in the Gaussian distribution, ranges from 1.4‰ to 16.5‰ (for countries with only 1 sample, 5‰ is assumed). The resulting global mean signatures, weighted by grid-level emission, become heavier over time (Figure S4) due to changes in emissions, especially the increased coal emissions from China that are associated with heavier signatures (-39.1‰ as a country mean) compared with other major coal-producing countries. A similar approach is also applied to ONG *δ*^13^C-CH_4_ signatures with the only exception to include temporal changes in the U.S. mean signatures (as discussed in section 2.2). For geological seeps emissions, 1.5‰ is assigned as the global weighted mean uncertainty based on the uncertainty estimates for four main categories of natural geo-CH_4_ emissions from Etiope et al. (2019).

The influence of the uncertainties of *δ*^13^C-CH_4_ signatures on emission partitioning are estimated using the mass balance equations of both CH_4_ and *δ*^13^C-CH_4_. Following SI-Eq.1 to SI-Eq.9, we conduct 10,000 MC simulations for each year by randomly sampling source signatures for each emission category from the constructed Gaussian distributions at grid-level. Emission scenario C_WL+ is used for the calculation to be consistent with the global weighted mean estimates from above. The global mean FE magnitude is estimated to be 167 Tg/yr, while the uncertainty is estimated to be 9.8 Tg/yr. The mass balance approach yields some inter-annual variability (without a statistically significant temporal trend) in the global mean FE magnitude (up to 50 Tg/yr) and the its uncertainty (range 9.2-10.4 Tg/yr); however, it is partially due to the propagation of inter-annual variability in atmospheric CH_4_ and *δ*^13^C-CH_4_ in the calculation, which does not reflect the real temporal variation in FE magnitude. Thus, a fixed 167 Tg/yr FE is assumed in many emission scenarios in this study. Note that this uncertainty of 9.8 Tg/yr only accounts for the uncertainty in source signatures by assuming no uncertainties in emissions, sinks, and atmospheric CH_4_ and *δ*^13^C-CH_4_ observations, which is the reason why this number is significantly smaller than the uncertainty estimates in Schwietzke et al. (2016). Uncertainties due to tropospheric Cl sinks, OH fractionation, WL areas and emissions, and geological seep emissions are evaluated separately in our study.
